# Single-Shot Multi-Stage Damage and Ablation of Silicon by Femtosecond Mid-infrared Laser Pulses

**DOI:** 10.1038/s41598-019-56384-0

**Published:** 2019-12-27

**Authors:** Kevin Werner, Vitaly Gruzdev, Noah Talisa, Kyle Kafka, Drake Austin, Carl M. Liebig, Enam Chowdhury

**Affiliations:** 1 The Ohio State University, Department of Physics, Columbus, OH 43224 USA; 2 BAE Systems, 130 Daniel Webster Hwy., MER15-1813, Merrimack, NH 03054 USA; 3 University of New Mexico, Department of Physics and Astronomy, Albuquerque, NM 87131 USA; 4 University of Rochester, Laboratory for Laser Energetics, Rochester, NY 14623 USA; 5 Air Force Research Laboratory, Materials and Manufacturing Directorate, Wright-Patterson Air Force Base, OH 45433 USA; 6 The Ohio State University, Department of Material Science and Engineering, Columbus, OH 43224 USA; 7 The Ohio State University, Department of Electrical and Computer Engineering, Columbus, OH 43224 USA

**Keywords:** Materials science, Optics and photonics, Physics

## Abstract

Although ultrafast laser materials processing has advanced at a breakneck pace over the last two decades, most applications have been developed with laser pulses at near-IR or visible wavelengths. Recent progress in mid-infrared (MIR) femtosecond laser source development may create novel capabilities for material processing. This is because, at high intensities required for such processing, wavelength tuning to longer wavelengths opens the pathway to a special regime of laser-solid interactions. Under these conditions, due to the λ^2^ scaling, the ponderomotive energy of laser-driven electrons may significantly exceed photon energy, band gap and electron affinity and can dominantly drive absorption, resulting in a paradigm shift in the traditional concepts of ultrafast laser-solid interactions. Irreversible high-intensity ultrafast MIR laser-solid interactions are of primary interest in this connection, but they have not been systematically studied so far. To address this fundamental gap, we performed a detailed experimental investigation of high-intensity ultrafast modifications of silicon by single femtosecond MIR pulses (λ = 2.7–4.2 μm). Ultrafast melting, interaction with silicon-oxide surface layer, and ablation of the oxide and crystal surfaces were *ex-situ* characterized by scanning electron, atomic-force, and transmission electron microscopy combined with focused ion-beam milling, electron diffractometry, and μ-Raman spectroscopy. Laser induced damage and ablation thresholds were measured as functions of laser wavelength. The traditional theoretical models did not reproduce the wavelength scaling of the damage thresholds. To address the disagreement, we discuss possible novel pathways of energy deposition driven by the ponderomotive energy and field effects characteristic of the MIR wavelength regime.

## Introduction

The field of intense mid-infrared (MIR) laser matter interaction has recently gained wide ranging interests due to novel phenomena like photon acceleration in metasurfaces^1^ to generation of attosecond pulses reaching carbon K-edge^2^, and demonstration of megafilamentation in atmosphere^3^, which may open doors to many exciting new applications^4^ and help probe novel phenomena^5^. Intentional MIR femtosecond laser-induced damage (fs-LID) can be used for the sub-surface micromachining of multi-layer materials, such as the fabrication of buried silicon waveguides operating in the telecommunications band^6^. Motivated by promise of such science and technology advances, there are several major efforts under way for the development of large MIR laser facilities such as the Extreme Light Infrastructure (ELI ALPS)^7^ in Europe or BESTIA (Brookhaven Experimental Supra-Terawatt Infrared at Brookhaven Accelerator Test Facility)^8^. The ability to accurately predict MIR fs-LID threshold (fs-LIDT) is crucial to the design and development of new sources^9^ in this wavelength regime. However, fs-LID and material modification studies performed so far have been mostly limited to near-infrared (NIR) wavelengths near 780–800 nm. Thus, at present, a significant gap remains in theoretical understanding and experimental data and results for fs-LID at MIR wavelengths^10,11^, which substantially slows down the fundamental and applied research in this area.

In parallel with this gap, our research is motivated by the fundamental fact that the dual nature of light supports two regimes of energy transfer from light to matter. First, light is absorbed by the small discrete energy portions referred to as photons. Second, as an electromagnetic wave, light supplies energy to the electron sub-system by periodically accelerating and decelerating electrons. These periodic electron dynamics are frequently referred to as laser-driven electron oscillations. These oscillations can be characterized by a cycle-averaged energy referred to as the ponderomotive energy. Under the single frequency approximation, the ponderomotive energy of an oscillating conduction-band electron:

1 $$\begin{array}{c}{W}_{{\rm{P}}}=\frac{{e}^{2}{E}_{0}^{2}}{4{m}_{{\rm{CB}}}{\omega }^{2}}\end{array}$$

is expressed via effective electron mass *m*_*CB*_; carrier laser frequency *ω*, and peak electric field *E*_0_ of the driving laser pulse. Eq. (1) assumes the amplitude of the laser-driven momentum oscillations is *eE*_0_*/ω*. Due to λ^2^ scaling in Eq. (1), the ponderomotive energy of high-intensity MIR pulses can exceed the energy of a single photon *ħω*, introducing new effects into the absorption of laser-pulse energy. In typical semiconductors, ultrafast absorption is coupled to three types of electronic excitations: inter-band transitions from valence to conduction band separated by a band gap of few eV; intra-band transitions within the same energy band; and transitions from the valence band to defect levels or from defect levels to the conduction band^12^. For interaction of good-quality crystals with sub-200-fs laser pulses, contributions of the defect-to-band and band-to-defect transitions can be neglected since they involve electron-defect interactions that require more time than the pulse duration^13,14^.

At ultraviolet (UV), visible, and NIR wavelengths, ${W}_{P}\ll \hslash \omega$ even at laser intensity as high as fs-LID and ablation thresholds. Under those conditions, intra-band excitation and free-carrier absorption^15^ as well as the inter-band transitions are associated with quantized variations of electron energy due to absorption of single or multiple laser photons. The inter-band excitation happens in the multiphoton regime since inter-band tunneling is negligible^17,18^ according to the Keldysh photoionization model^16^. This regime can be referred to as *photon-driven*.

In the opposite limit of ${W}_{P}\gg \hslash \omega$, the photon energy is small enough to provide quasi-continuous variations of electron energy. As a criterion, one can consider photon energy of 0.04 eV corresponding to $\lambda \ge 25$ μm that is the room-temperature electron energy^19^. For this *field-driven* absorption regime, tunneling ionization dominates in inter-band excitation^16^, and deposition of ponderomotive energy via electron-particle collisions becomes a major mechanism of light absorption. Specific features of tunneling ionization^20^ suggest that the intra-band absorption of the ponderomotive energy dominates in the overall energy transfer from light to the electrons^19^.

Therefore, a transition from the quantized photon-driven to continuous field-driven regime of absorption is expected in the wavelength range from 1 to 25 μm (corresponding photon energy is 1 eV – 0.04 eV) for typical semiconductors. Studies of wavelength scaling of fs-LID (permanent modification) or ablation (material removal) thresholds can substantially assist in a search for the fundamental transition between the two absorption regimes.

Experimental data and theoretical results^11,17,18^ suggest an increasing LIDT with increasing wavelength in the *photon-driven* regime. An ultimate threshold of the field-driven breakdown is that by DC electric field in a semiconductor^21^, but it is orders of magnitude lower than peak time-dependent field at fs-LIDT^11,18,22–28^. Therefore, it is reasonable to attribute the transition between the photon-driven and field-driven regimes to a maximum of wavelength scaling of fs-LIDT at some wavelength in the range 1 to 25 μm.

**Figure 1:**
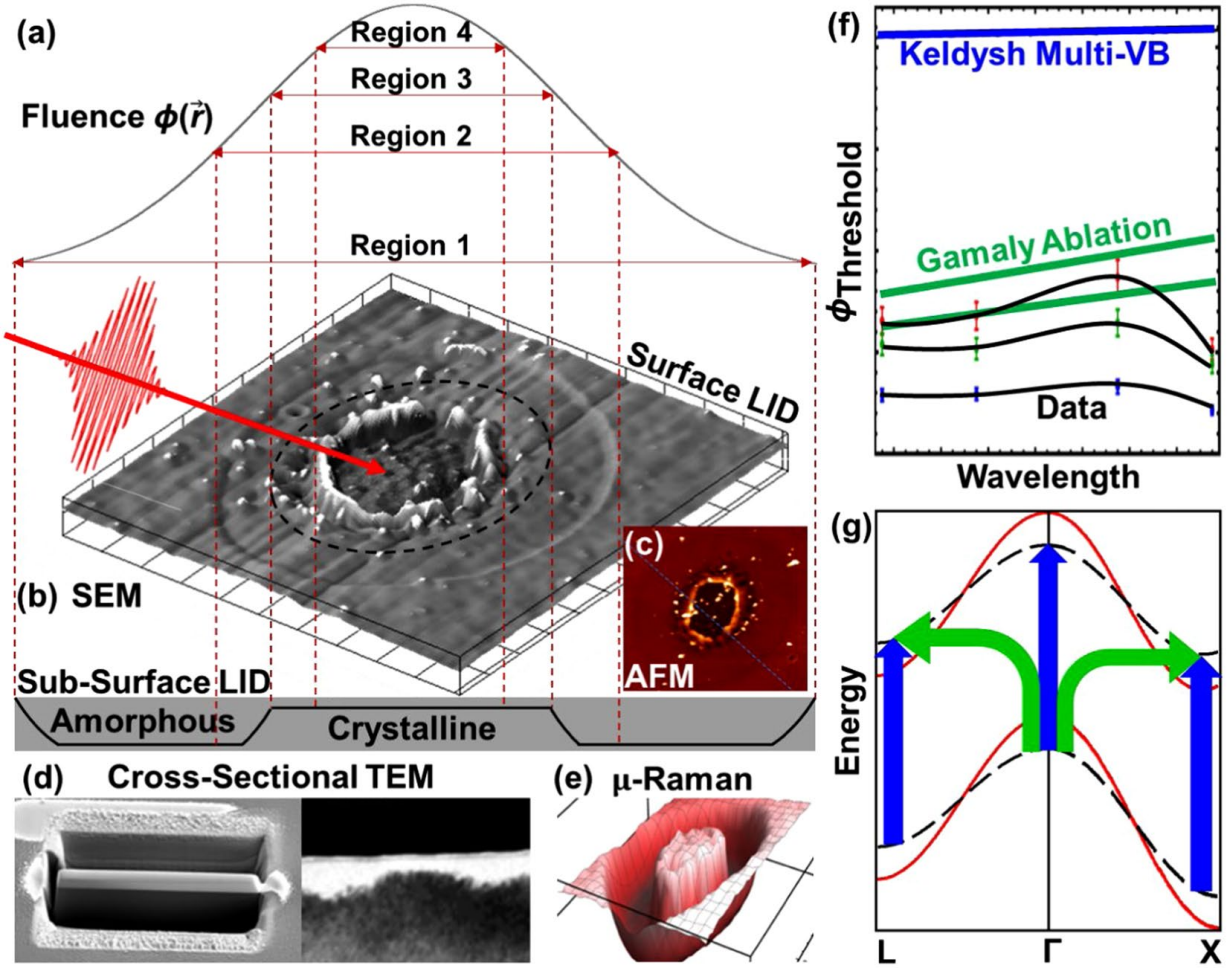
A schematic workflow of the research includes (**a**) damaging of a silicon surface by a single ultrashort laser pulse with Gaussian space distribution of fluence; detection and characterization of the four regions of a damage spot (see description in the text) by (**b**) SEM, (**c**) AFM, (**d**) cross-sectional TEM, and (**e**) μ-Raman spectroscopy; (**f**) measurement of wavelength scaling of the thresholds of formation of each characteristic damage-spot region followed by fitting of the scaling with the Keldysh and Gamaly models; and (**g**) theoretical analysis of competing direct (vertical blue arrows) and indirect (bent green arrows) inter-band electron transitions between initial (solid red) and laser-disturbed (black dashed) energy bands.

In this work, we present a systematic study of irreversible ultrafast MIR laser interactions with the single crystal silicon surface (Fig. 1). Multiple stages of fs-LID and ablation of single-crystal silicon by MIR femtosecond pulses are reported, and scaling of single-pulse thresholds of each individual stage as a function of laser wavelength is measured experimentally, demonstrating a pronounced maximum in the explored MIR part of the optical spectrum. These observations show a significant qualitative difference between the strong-field ultrafast laser-solid interactions at MIR and those at UV, visible, and NIR wavelengths, for which absorption is assumed to be photon-driven^11,18,22–24,27,29–33^. Estimations of the Keldysh parameter demonstrate that a transition from multiphoton to tunneling regime of the photoionization happens at shorter wavelengths than the position of the damage or ablation threshold maximum. Therefore, the maximum of the wavelength scaling of ablation and LID threshold is reached under domination of the tunneling ionization and cannot be attributed to the transition between the two photoionization regimes. Two traditional models of laser-induced damage based on the Gamaly approximation^29^ and the Keldysh photoionization theory^16^ fail even to qualitatively reproduce the wavelength scaling of the damage/ablation threshold. Qualitative analysis of involved mechanisms suggests attributing the experimental data to the transition from the photon-driven to field-driven regimes of absorption that is substantially influenced by a competition between different inter-band excitation paths and laser-induced modification of band structure of silicon^34^.

## Results

**Figure 2:**
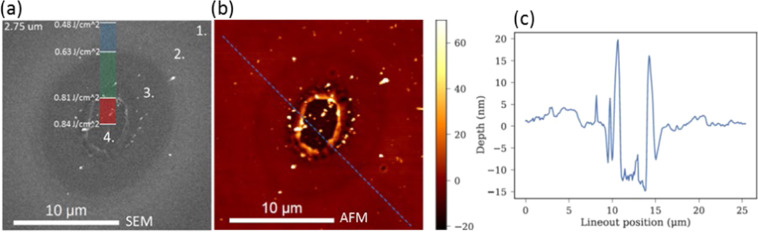
Side-by-side SEM (**a**), AFM (**b**), and AFM lineout (**c**) of the same damage spot produced at fluence of 0.84 J/cm^2^ at wavelengths 2.75 μm. Regions of interest 1–4 are described in the main text. SEM overlay of panel (**a**) shows local fluence. AFM lineout of panel (**c**) is taken along the blue dotted line shown in the panel (**b**).

**Figure 3:**
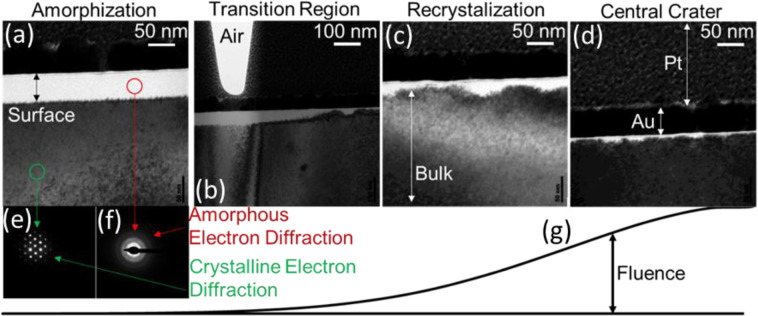
Cross-sectional TEM images (**a**–**d**) of the sub-surface damage below the same site featured in Fig. 2. Four different regions of the same damage site are shown: (**a**) region 2 (amorphization occurs), (**b**) between regions 2 and 3, (**c**) region 3 (recrystallization occurs), and (d) region 4 (crater). The sacrificial platinum layer, protective gold layer, laser-modified surface region, and bulk silicon substrate are indicated. Electron diffraction of the darker contrast areas show a crystalline signal (**e**) while bright white areas indicate amorphization (**f**). The local incident laser fluence increases from left to right for each TEM image; i.e. images further towards the right are closer to the central crater (**g**). TEM was performed at the Center for Electron Microscopy and Analysis (CEMAS) at The Ohio State University.

**Figure 4:**
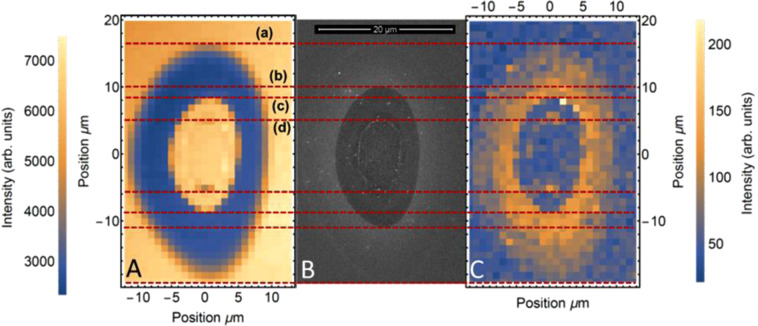
μ-Raman spectroscopy (**A**,**C**) of a damage spot produced at fluence of 0.87 J/cm^2^ at wavelength 3.5 μm. Peak intensity of the 520 cm^−1^ signal (**A**) shows crystalline phase, while the 480 cm^−1^ signal (C) shows amorphous phase. An SEM image of the spot (**B**) is shown for proper identification of different regions of the damage spot. Dotted lines (a–d) indicate the different regions of surface or subsurface LID discussed in the main text. μ-Raman spectroscopy measurements were performed at the US Air Force Research Laboratory in Dayton, Ohio.

Single-pulse MIR fs LID on single-crystal silicon (see Methods) was characterized *ex-situ* by scanning electron microscopy (SEM), atomic force microscopy (AFM), cross-sectional transmission electron microscopy (TEM), and $\mu$-Raman spectroscopy (Figs. 1–4).

Figure 2 shows representative SEM and AFM images of a damage spot. Three characteristic regions of surface modification were observed, and Fig. 2a shows the approximate local fluence within each of the regions. The SEM reveals a central ablation spot, surrounded by a distinct crater rim (region 4 in Fig. 2a). Region 3 in Fig. 2a is enclosed by the middle ring and shows a similar contrast to the central crater. The third ring, barely visible, encloses region 2 (Fig. 2a), and exhibits a change in contrast compared to the other two regions. Region 1 in Fig. 2a has no outer boundary, and represents the undamaged silicon substrate with no permanent changes detected via SEM or AFM *ex-situ*. In the AFM spatially resolved depth profile of the same damage site (Fig. 2b,c), the same 3 rings are all visible, each with the same lateral dimensions as in the SEM images (Fig. 2a). The height profile from the AFM images (Fig. 2c) shows that region 2 is slightly raised over regions 3 and 1, and confirms that area 4 is an ablation crater. Extra SEM images (see Supplementary Materials) suggest that the chaotic nature and nano-roughness within the shorter wavelength damage site is far more pronounced compared that of the longer wavelength, for a fixed fluence.

Figure 3 depicts representative sub-surface structure of the four regions of the damage spot shown in Fig. 2. The topmost layer of the sample shown with a white color has amorphous structure as identified by electron diffraction (Fig. 3f). Deeper layers of darker colors have crystalline structure (Fig. 3e). Within the resolution limit of the imaging device, no polycrystalline areas could be found. Figure 3d presents an image of the sub-surface layers of the innermost ring (region 4 in Fig. 2), where ablation formed the crater. A relatively non-uniform 20–25 nm thick amorphous layer is observed in the middle ring (region 3 in Fig. 2). The region bound by the barely visible outermost ring has a relatively thick (50 nm), surprisingly uniform amorphous layer. In each image of Fig. 3, a very thin (1–2 nm thick) native oxide layer is visible on the sample surface in the form of a slight contrast change. Extra TEM data (see supplementary Materials) suggest that the middle ring and the onset of very thick amorphization do not occur in the same location. The middle ring boundary has a lower fluence threshold than the crystalline/amorphous boundary. The crystalline/amorphous boundary occurs ~2 um closer to the center than the outer ring.

Spatially resolved micro-Raman spectroscopy of a typical damage site (Fig. 4) was performed at the 520 cm^−1^. Raman peak associated with the crystalline phase (Fig. 4a) and the 480 cm^−1^. Raman peak attributed to amorphous structure (Fig. 4c). The micro-Raman images match up with a SEM image of the damage spot (Fig. 4b) to properly attribute the crystalline and amorphous contents to the specific parts of the damage spot. Red dotted lines are aligned to certain transitions in either the Raman mapping or the SEM. Line (a) shows the transition from the highly amorphous region to the un-damaged sample Line (b) is aligned to the middle ring of the SEM image within the highly amorphous region. On the Raman mapping, this ring is not distinguished, indicating that there is no significant change in crystallinity below this ring. Line (c) is aligned to the transition between highly amorphous and highly crystalline signals on the Raman mapping. This transition occurs several um closer to the center than the middle ring. Line (d) shows that the regions both inside and outside the rim show a strong crystalline signal. Overall, the Raman mapping is consistent with the TEM images of Fig. 3.

**Figure 5:**
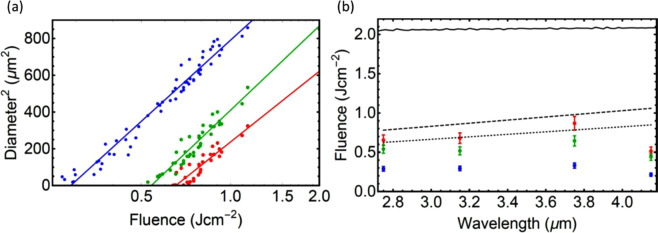
Scaling of area of laser-modified spot on the surface with peak laser fluence at wavelengths of 2.75 μm (**a**) to illustrate the approach of evaluation of threshold for phase explosion (red), ablation (green), and melting (blue) by extrapolating a linear fit towards zero area. (**b**) Wavelength scaling of LID threshold as obtained from experimental data (dots with error bars), Gamaly model (dotted line – for indirect band gap; dashed line – for direct band gap), and two-band Keldysh model (solid).

Figure 5a illustrates the approach to the measurement of LID thresholds for the three most probable damage effects under consideration: phase explosion (center of a LID spot), ablation, and ultrafast melting (most outer part of the spot). The diameter of each ring, on each damage site, for each wavelength, was measured from SEM images. The data were then plotted as dependence of diameter squared on logarithm of peak fluence to determine LIDT (Fig. 5a) for each region. Scaling of the damage thresholds with wavelength are plotted in Fig. 5b. Damage thresholds remain constant or rise slightly with increasing wavelength, except for the longest wavelength where a drop in damage thresholds occurs for every regime. A comparison of the experimental wavelength scaling to the predictions of the Gamaly^29^ and Keldysh^16^ models is shown in Fig. 5b (see details of the simulations in Materials and Methods and in Supplementary Materials). The Gamaly model^29^ qualitatively agrees with the strong ablation experimental data and even captures the overall trend for much of the data at shorter wavelengths for indirect band gap, but fails to capture the reduction in LIDT for the longest wavelength. The simulations with the Keldysh photoionization model^16^ greatly overestimate the experimental LIDTs and do not even qualitatively reproduce the experimentally observed trend.

## Discussion

SEM (Fig. 2), AFM (Fig. 2), and TEM (Fig. 3) images as well as the Raman spectra mapping (Fig. 4) suggest three stages of surface MIR laser damage each characterized by a well-defined threshold (Fig. 5a). Formation of amorphous silicon in the outermost region indicates that the velocity of the cooling front exceeds the critical speed of amorphization in silicon (12–25 m/s)^22^. This region raises slightly above the sample surface and exhibits significant uniformity of the amorphous-layer thickness attributed to the Gaussian space distribution of fluence. These features, along with the measured threshold, are consistent with those of the ultrafast melting at NIR wavelengths^35,36^, except the reduced thickness (30 nm for NIR versus 50 nm for MIR) attributed to strong linear absorption of the NIR photons. Therefore, it is reasonable to assume that the ultrafast melting induced at MIR wavelengths is driven by the mechanism established for the NIR wavelengths: crystal-lattice instability by increase of free-carrier density to approximately 10% of the valence band electron density^36–40^, i.e., about 10^22^ cm^−3^. It is believed the energy of the excited electrons does not substantially affect that process^38,39^.

The region bound by the middle circle demonstrates the features associated with the ultrafast melting and amorphization, but several nm of material are removed through spallative ablation. A similar phenomenon was observed at MIR wavelengths on Ge^10^ and at NIR wavelengths on Si^35^. However, at NIR wavelengths, typically only the top oxide layer is removed in this zone^35^. In contrast, MIR wavelengths show removal of both the oxide layer and some underlying material.

Both the cross-sectional TEM (Fig. 3) and the $\mu$-Raman mapping (Fig. 4) show a transition from amorphous to crystalline material several microns inside the middle ring. For comparison, at NIR wavelengths, a strong amorphous phase persists even partially inside the crater rim^35^. Furthermore, the single-crystal recrystallization revealed by TEM (Fig. 3) also differs from the NIR case, where polycrystalline phase was observed in the form of hillock formation within the crater rim^35^. The difference between the amorphization by MIR and by NIR pulses^35,41,42^ is partly justified by the fact that the MIR experiments were performed on a (100) surface while the NIR experiments were reported for a (111) crystalline surface. It has been shown that a (111) crystal surface is amorphized more readily after laser-induced melting than the (100) surface^41–43^, while the transition from amorphization to crystallization occurs at a lower threshold fluence on a (100) surface compared to (111). Moreover, (111) surfaces were found to recrystallize into a polycrystalline phase at higher fluences when compared to (100) surfaces.

The specific band structure of silicon^34^ is favorable for a variety of laser-stimulated microscopic process that may contribute to the surface modification. The most rigorous approach to interpretation of the reported above data should consider ab-initio simulations of the ultrafast strong-field MIR interactions^44^. However, available ab initio simulations miss a proper incorporation of electron-particle collisions and can be technically done over 10–20 femtoseconds of the laser-solid interaction^45^. Other numerical models^16,26,29,33,38,39,46,47^ are not valid for analysis of the high-intensity MIR laser-silicon interactions because they use the approximations that do not fit the indirect-gap band stricture of silicon. For these reasons, we limit our discussion to qualitative analysis of the following relevant items: A) inter-band electron excitation; B) absorption mechanisms; C) microscopic criteria of ablation threshold, and D) transient modifications of band gaps.

Prior to the analysis of microscopic mechanisms, we note that contribution of the native oxide layer to the ablation can be neglected in spite of its significant absorption around 2.6–2.8 μm and 4.4 μm^48,49^. A primary ablation of the layer would be expected if it appreciably contributed to the energy deposition. However, structure of the ablated spot (Figs. 2 and 3) suggests that the surface layer was blown up from bottom at each tested wavelength. A reasonable interpretation of that specific ablation morphology should consider a dominant electron excitation and absorption of laser-pulse energy in sub-surface layers of crystalline silicon.

**Figure 6:**
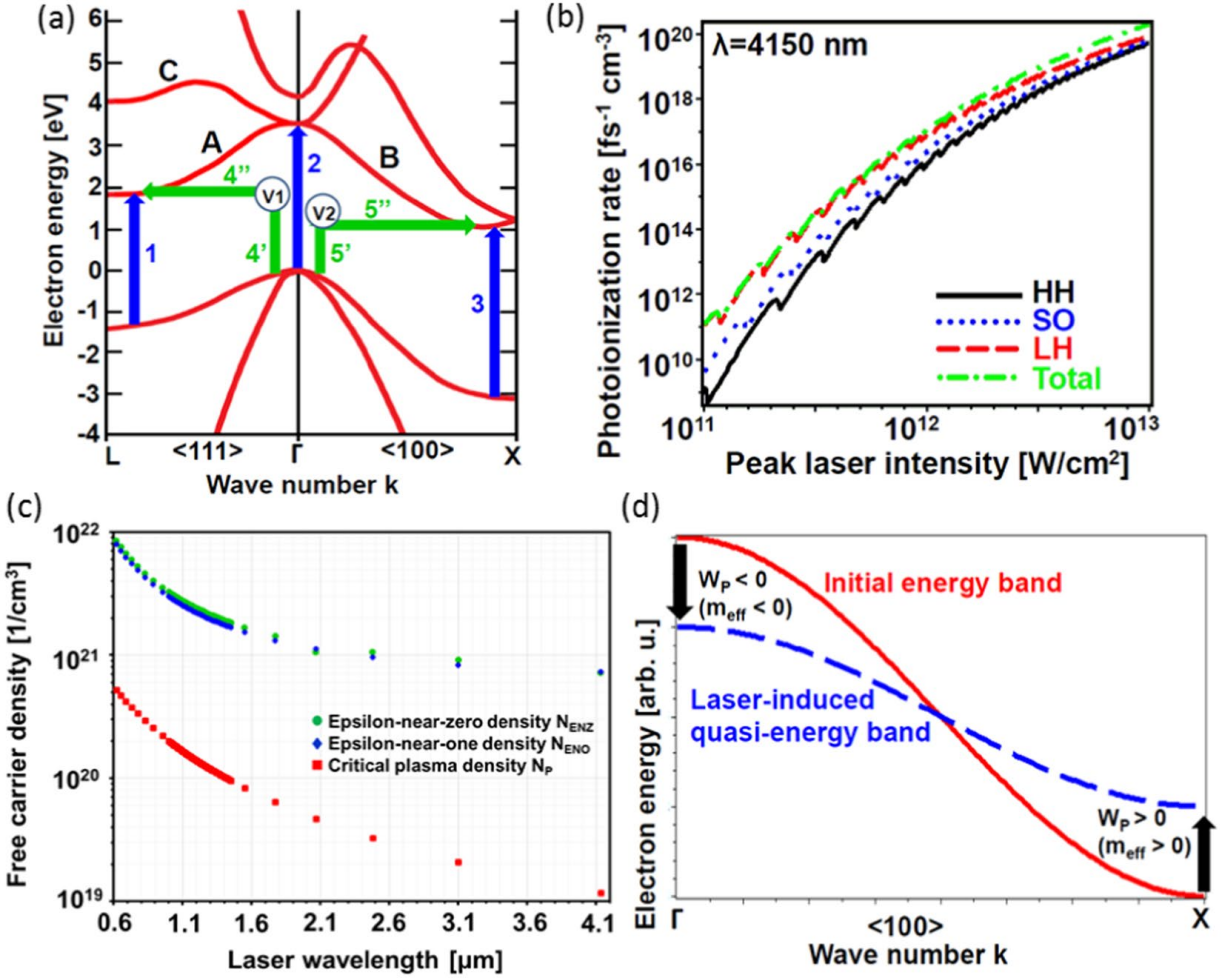
(**a**) A sketch of competing direct (blue vertical arrows) and indirect (bent green arrows) inter-band electron transitions contributing to the nonlinear absorption and electron-hole plasma generation. V1 and V2 depict the two virtual states involved in the indirect transitions. (**b**) Scaling of the photoionization rate with peak laser intensity at wavelength of 4150 nm to illustrate similarity of contributions of the heavy-hole (HH – black solid), light-hole (LH – red dashed), and split-off (SO – blue dotted) valence bands to the total rate of the inter-band electron excitation (green dash-dotted). (**c**) Wavelength scaling of critical plasma *N*_*P*_ (red squares), epsilon-near-zero *N*_*ENZ*_ (green dots), and epsilon-near-one *N*_*ENO*_ (blue diamonds) densities evaluated by equations of section 7 of Supplementary materials for *m*_*eff*_ = 0.18 of free-electron mass and Drude-model collision time *τ*_*D*_ = 1 fs. (**d**) A sketch of laser-induced reduction of the B branch of conduction-band bandwidth along (100) direction due to modification of the initial band (red solid) by ponderomotive energy *W*_*P*_ of laser-driven electron oscillations. *W*_*P*_ is negative in the center of the Brillouin zine due to negative effective mass, and positive at the zone edge due to positive effective mass near the X valley.

The major processes of energy deposition in crystalline silicon are influenced by the specific structure of indirect-gap energy bands^34^. Since amplitude of the laser-driven variations of electron momentum *eE*_0_*/ω* is 3–5 times larger than the half-width of the first Brillouin zone at threshold fluence, contributions to the inter-band transitions may simultaneously occur in the vicinity of Γ, X, and L points^34^ of the silicon band structure. In spite of complicated nature of those processes, some substantial physics of the ultrafast electron excitation and associated absorption can be delivered by simple estimations of characteristic parameters with proper values of energy gaps and effective masses (Fig. 6a)^34^.

A dominating regime of the laser-driven direct inter-band electron excitation (transition paths 1, 2, and 3 in Fig. 6a) can be identified by evaluation of the Keldysh adiabaticity parameter^16^:

2 $$\begin{array}{c}\gamma =\frac{\omega \sqrt{m\Delta }}{eE}\end{array}$$

where *m* is a proper value of reduced effective electron-hole mass, and Δ is an energy gap between involved valence and conduction bands at the characteristic band-structure points. Estimations for the Γ point (effective electron mass is 0.188 of free-electron mass *m*_*e*0_; direct band gap is 3.28 eV^34^) are summarized in Table 1. At all wavelengths, the values of the Keldysh parameter are well below 1.0 at the Γ and X points and suggest domination of the tunneling regime for the direct inter-band transitions (see Supplementary Materials). Parameters of the L valley support the multiphoton regime of the inter-band transitions, the rate of which is several orders of magnitude lower than the rate of the tunneling transitions (see Supplementary Materials). Therefore, major contributions to the direct inter-band promotion of valence electrons is expected from the Γ and X points of the band structure.

**Table 1:** Laser wavelength λ; photon energy *ħω*; peak fluence *F*_*ABL*_*/F*_*MELT*_, peak intensity *I*_*ABL*_/*I*_*MELT*_, and peak electric field *E*_0*ABL*_/*E*_0*MELT*_ of laser pulses at threshold of ablation/melting. For the ablation/melting thresholds, there are shown the Keldysh parameter *γ*_*ABL*_ and *γ*_*MELT*_ (for the direct transition from the LH valence to the conduction band at the Γ point); the Franz-Keldysh reductions of band gap δΔ_*ABL*_ and δΔ_*MELT*_ for LH valence band; ponderomotive energy *W*_*pABL*_ and *W*_*pMELT*_ of the conduction-band electron oscillations at the Γ point; and ratios *W*_*pABL*_*/ħω* and *W*_*PMELT*_*/ħω* of the ponderomotive energy to photon energy.

λ [nm]	*ħω* [eV]	*F*_*ABL*_*/F*_*MELT*_ [J/cm^2^]	*I*_*ABL*_/*I*_*MELT*_ [TW/cm^2^]	*E*_0*ABL*_/*E*_0*MELT*_ [V/nm]	*γ*_*ABL*_*/γ*_*MELT*_	δΔ_*ABL*_/δΔ_*MELT*_ [eV]	*W*_*pABL*_/*W*_*pMELT*_ [eV]	*W*_*pABL*_*/ħω/W*_*PMELT*_*/ħω*
2750	0.45	0.65/0.29	2.56/1.13	2.37/1.57	0.35/0.53	1.75/1.33	2.80/1.23	6.20/2.73
3150	0.39	0.68/0.29	2.66/1.15	2.42/1.59	0.30/0.46	1.77/1.34	3.82/1.65	9.69/4.19
3750	0.33	0.87/0.33	3.40/1.29	2.73/1.69	0.23/0.37	1.92/1.40	6.93/2.64	20.92/7.97
4150	0.23	0.52/0.21	2.01/0.84	2.11/1.36	0.26/0.41	1.62/1.21	5.03/2.09	16.82/6.99

The specific indirect-gap band structure of silicon also favors indirect inter-band transitions (Fig. 6a). They can be considered as multiphoton excitation of a valence electron to a virtual state V1 (for the Γ-to-L transition) or V2 (for the Γ-to-X transition) followed by phonon interaction with the excited electron to promote it to either L or X valley of the conduction band^12,19,34^. Rates of the indirect transitions around the Γ point (see Supplementary Materials and Methods) can be estimated using the lifetime *δt*_*i*_ (*i* = V1 or V2) of the virtual states from the uncertainty relation^20^:

3 $$\begin{array}{c}\delta {t}_{i}\cdot \delta {W}_{i}\ge \hslash \end{array}$$

by assuming the energy uncertainty *δW*_*i*_ is as much as the indirect band gaps (i.e., 1.12 eV for the Γ-to-X transition and 2.0 eV for the Γ-to-L transition^34^). Ratio of the total probabilities *P*_4_ and *P*_5_ of the indirect transitions (arrows 4 and 5 in Fig. 6a) to the probability *P*_2_ of the direct inter-band excitation (arrow 2 in Fig. 6a) can be evaluated via the direct-transition rate *w*_*MP2*_ by the Keldysh formula^16^, multiphoton-transition rates *w*_V1_ and *w*_V2_ for the direct excitation of valence electrons to the virtual states, and the time *τ*_eph_ of electron collisions with polar phonons^12,19^ (see Methods):

4 $$\begin{array}{c}\frac{{P}_{5}}{{P}_{2}}=\frac{{w}_{{\rm{V}}2}\delta {t}_{{\rm{V}}2}}{{w}_{{\rm{MP}}2}{\tau }_{{\rm{eph}}}},\frac{{P}_{4}}{{P}_{2}}=\frac{{w}_{{\rm{V}}1}\delta {t}_{{\rm{V}}1}}{{w}_{{\rm{MP}}2}{\tau }_{{\rm{eph}}}}.\end{array}$$

Estimations (see Supplementary Materials) suggest that the ratio of the transition rates of Eq. (4) is at least of the order of 10^1^–10^2^ for V1 and 10^2^–10^3^ for V2 at wavelength 2.75 μm. The smallest reported electron-phonon collision time measured at NIR laser wavelengths is about 30–60 fs^50,51^. However, that collision time shows a strong dependence on energy of conduction-band electrons^52^. Small photon energy characteristic of MIR wavelengths is favorable for generation of the conduction-band electrons with energy of the order of 0.1 eV (Table 2). In this case, the electron-phonon collision time is as large as few hundreds of femtoseconds^52^. Therefore, the ratio of the virtual-level lifetime *δt*_*V1*_ and *δt*_*V2*_ (about 0.5–1.0 fs) to *τ*_eph_ is of the order of 0.001. Equation (4) suggests that the rates of the indirect excitations from Γ to X and L valleys are similar to or higher than the rate of the direct transitions at the Γ-point at 2.75 μm. With increase of wavelength, the ratio of the transition rates reduces by few orders of magnitude because the tunneling regime of photoionization exhibits very weak wavelength scaling^16^, but the rate of the multiphoton excitation to the virtual levels V1 and V2 substantially decrease with reduction of photon energy (see Supplementary Materials). Therefore, within the MIR regime, a significant contribution of the indirect Γ-to-X transitions to the overall free-carrier generation at shorter wavelengths is replaced by domination of the direct inter-band tunneling excitation at the longer wavelengths.

**Table 2:** Laser wavelength at spectrum peak λ; photon energy at the laser wavelength *ħω*; number of laser photons to bridge the indirect band gap for Γ-to- X transitions *N*_*ind*_ = < *Δ*_*ind*_/*ħω* + 1 > *(Δ*_*ind*_ = 1.12 eV^34^); excessive energy *δ*_*ind*_ = *N*_*ind*_ *ħω – Δ*_*ind*_ of the electrons arriving to the X valley; number of laser photons to bridge the direct band gap at the Γ point *N*_*dir*_ = < *Δ*_*dir*_/*ħω* + 1 > (*Δ*_*dir*_ = 3.28 eV^34^); and excessive energy *δ*_*dir*_ = *N*_*dir*_ *ħω – Δ*_*dir*_ of the electrons generated at the Γ point.

λ [nm]	ħω [eV]	N_ind_	δ_ind_ [eV]	N_dir_	δ_dir_ [eV]
2750	0.45	3	0.23	8	0.32
3150	0.39	3	0.05	9	0.23
3750	0.33	4	0.20	10	0.02
4150	0.23	5	0.03	15	0.17

An attempt to evaluate the rate of the Γ-point direct inter-band electron transitions by the Keldysh formula^16^ applied to three valence bands (Fig. 6b and Supplementary Materials) delivers the total rate about 10^17^ 1/(fs cm^3^). Therefore, the total density of the laser-generated electron-hole pairs is of the order of 10^19^ 1/cm^3^ by the end of a laser pulse according to the Keldysh model. This is two to three orders of magnitude below the density required to initiate the ultrafast melting^37,38^. This difference can result from the contribution of the indirect inter-band transitions that are neglected in the original Keldysh model^16^, but also from the simplified energy-momentum relation for the central part of the Brillouin zone utilized in the Keldysh formula^16^.

Since the duration of a single oscillation cycle of conduction-band electrons (9.17 fs at 2750 nm to 13.83 fs at 4150 nm) is almost one order of magnitude smaller than the characteristic time of momentum de-phasing by electron-phonon collisions at the bottom of the conduction band of silicon^52^, the collisions can be considered as a minor perturbation to the laser-driven oscillatory intra-band electron dynamics. This approach implies that the oscillating electrons and holes possess some ponderomotive energy of the oscillations that is retained in the free-electron sub-system due to the collisions. Estimated ponderomotive energy at the Γ point (Table 1) substantially exceed the photon energy. Moreover, at the ablation threshold, the ponderomotive energy exceeds direct band gaps, bandwidth of the lowest conduction bands of silicon (about 2. 7 eV), and even electron affinity of crystalline silicon (about 4.05 eV^34^). The latter fact means the conduction-band electrons pumped by the ponderomotive energy can be effectively emitted from the crystal to produce local violation of electric neutrality^53,54^ that is favorable for a spallation regime of ablation. Moreover, enhancement of the tunneling mechanism of the electron photoemission with reduction of driving laser frequency^55^ may make an appreciable contribution to reduction of the ablation threshold with increase of laser wavelength. Therefore, the collision-driven deposition of ponderomotive energy can be a more effective ultrafast mechanism of energy transfer from laser pulses to the crystal than absorption of single or multiple photons via the electron-photon-phonon collisions.

Several microscopic criteria have been reported to simulate ablation or damage threshold^27–33,37,56,57^ including critical energy, critical free-carrier plasma density *N*_*P*_, free-carrier plasma-resonance density, epsilon-near-zero (ENZ) free-carrier density *N*_*ENZ*_, epsilon-near-one (ENO) free-carrier density *N*_*ENO*_, phonon-softening plasma density, and crystal-lattice-instability plasma density (see Supplementary Materials, section 7). The Gamaly model^29^ based on deposition of critical amount of energy was employed to simulate the measured wavelength scaling of ablation and modification thresholds (see Fig. 5), but it did not qualitatively reproduce the scaling trend. It is notable that the critical-energy ablation criterion of the Gamaly model^29^ is based on a rough estimation of energy balance that neglects some important microscopic processes by assuming instant transitions of virtually all valence electrons to the conduction band and formation of solid-density plasma. In case of high-rate one-photon absorption, this assumption may be reasonable, but it is evidently violated at small rates of nonlinear absorption characteristic of the MIR wavelengths. Therefore, the process of energy transfer from light to the electrons of a solid is extended in time and supports “leakage” of the transferred energy to other process not incorporated into the model.

The microscopic ablation criteria based on critical free-carrier plasma density^17,22,31,33,46^, ENZ, and ENO free-carrier density^36,56,57^ employ the critical density of electron-hole plasma that is reached when the plasma frequency equilibrates with laser frequency. Those criteria assume the plasma is quasi-equilibrium and its critical density scales as λ^−2^ with laser wavelength (Fig. 6c). That scaling predicts reduction of those critical plasma densities by more than one order of magnitude with a transition from NIR to MIR wavelengths (Fig. 6c). It is remarkable that those criteria are based on the plasma critical density (that is about 10^19^–10^20^ cm^−3^ in the MIR wavelength range) that is well below the electron density (about 10^22^ cm^−3^) required to initiate the ultrafast melting^36–40^. Moreover, during the first 100–200 fs in silicon, experiments have shown the reflectivity change remains less than 10% even at fluence 1.5 times the melting threshold^58^. The weak variations of refractive index during a 200-fs laser pulse^10^ signals that the laser-generated electron-hole plasma is substantially transparent. This could occur if the plasma is, for example, far from equilibrium^59,60^. This hypothesis is further supported by estimations of the energy of laser-generated conduction-band electrons (Table 2) that suggest the electron-particle collision time is of the order of few hundreds of femtoseconds^52^. This special feature of the MIR wavelength range is favorable for generation of the non-equilibrium free-carrier plasma at the bottom of the conduction band. Therefore, the criteria based on the concept of plasma critical density may not be valid at the MIR wavelengths. In contrast, silicon excitation at visible and NIR wavelengths produces conduction-band electrons with average energy about few eV^37^. At that energy, electron-particle collision time is of the order of 1 fs^52^ and provides ultrafast equilibration of the free-carrier plasma. Therefore, the critical-plasma density criteria including ENZ and ENO criteria (see supplementary Materials) are more reasonable at shorter wavelengths.

To identify an adequate microscopic criterion of MIR fs-LID and ablation thresholds, we note that ultrafast melting and ablation of a solid should be attributed to weakening or breaking inter-atomic bonds^36–40^. Those bonds are provided by valence electrons. Therefore, weakening of the bonds should be associated with excitation the valence electrons to the conduction band^36–40^. Then, a reasonable criterion for the LID and ablation thresholds by ultrashort laser pulses should consider the electron density required to induce crystal-lattice instability via weakening of the inter-atomic bonds^10,36,37^. This density is obviously an intrinsic property of a crystal and must be a function of material rather than laser-pulse parameters. The λ^−2^ scaling of the ENZ, ENO, and critical-plasma densities (Fig. 6c) contradicts this conclusion and suggests that those criteria assume reduction of the density of broken or weakened bonds by more than one order of magnitude with the transition from NIR to MIR wavelengths. There is no a reasonable justification of this strong influence of laser wavelength on the intrinsic property of silicon.

The high values of threshold intensity (Table 1) imply significant transient modifications to the band structure of silicon, i.e., distortions of the shape of the bands^61^ and variations of the band gaps. There are several mechanisms of those modifications. For example, a phenomenon similar to the dynamic Franz-Keldysh effect^44,62^ reduces band gap. Since the high-intensity limit of that effect^44^ is like the one for dc electric fields^63^, the band-gap reduction can be estimated as follows^63^:

5 $$\begin{array}{c}\delta \Delta ={[{(e{E}_{0})}^{2}\frac{{\hslash }^{2}}{{m}_{{\rm{RE}}}}]}^{\frac{1}{3}},\end{array}$$

where *m*_RE_ is the effective reduced electron-hole mass in the direction parallel to electric field *E*_0_. Estimations for <111> direction around the Γ point suggest very substantial reduction of the band gaps of silicon both at the ablation and at the melting thresholds. Moreover, significant wavelength dependence of that effect^62^ suggests an enhancement of its contribution for the longer-wavelength of the tested MIR range.

The fundamental properties of silicon are favorable for several other effects that also modify the band gaps^21^. First, the top valence band and the lowest conduction band of silicon originate from the same hybrid atomic state^12,19,64^, those two energy bands are coupled. That coupling enhanced by electric field of laser radiation^64–66^ results in reduction of the band gaps^64–66^. However, its contribution cannot be estimated in a simple way and requires extended numerical simulations.

Second, band-gap shrinkage results from increase of the conduction-band population *N* according to the *N*^1/3^ law^66–68^. It reduces the band gaps by 1–2 eV at the conduction-band electron density 10^21^ 1/cm^3^and appreciably contributes to the overall modification of band structure^21^.

Third, generation of the conduction-band electrons makes another significant contribution to the band-gap modification by the band filling effect^66–68^. That modification results from the dominating filling of the lowest-energy conduction states that favors absorption of one extra photon to promote the newly arriving electrons to higher-energy vacant states of the band^21^. However, the influence of the ultrafast band-filling effect may be smaller compared to the usual estimations^66–68^ because of effective re-distribution of the electrons over almost entire conduction band due to the laser-driven oscillations^21^. Also, this effect can be substantially compensated by the band-gap shrinkage effect^37^.

Finally, the ponderomotive energy substantially contributes to the band-gap modification^16,61,69^. According to Eq. (1), that energy can make either positive or negative contribution to the band-structure modification depending on sign of effective mass at specific parts of the energy bands. For example (Fig. 6d), the X valley of the conduction band (Fig. 6a) is up-shifted due to positive effective electron mass while the Γ-point segment of the band is down-shifted because of the negative effective mass near the Γ point. Such ponderomotive-energy effects reduce bandwidth of the energy bands, remove degeneracy of the bands, and can produce very prominent modification of the band gaps. The small value of energy of the conduction electrons (Table 2) generated by MIR wavelengths suggests extended electron-particle collision time^52^ that is favorable for this mechanism of band-gap modification.

The special feature of silicon is that all those effects simultaneously contribute to the band-structure modification by either band-gap reduction (e. g., the analog of the dynamic Franz-Keldysh effect, band-gap shrinkage, and enhancement of band coupling) or band-gap increase (band filling and ponderomotive energy) making overall dynamics of the band-structure modification very complicated. Moreover, the ultrafast dynamics driven by the femtosecond laser pulses can be appreciably influenced by quantum interference between different paths of the laser-driven electron excitations. Also, the effects of the non-parabolic, non-Kane conduction band topology may be very important. Time-dependent ab initio simulations are the most appropriate to attack that complicated problem, but the currently available approaches cannot properly address those effects^45^.

## Conclusions

Single-shot fs-LIDA experiments on silicon followed by cross-sectional TEM, SEM, AFM, and micro-Raman spectroscopy have revealed unique LIDA morphology attributed to multiple stages of surface LIDA. For certain well defined fluences, a “weak” spallative ablation occurs which was not observed for NIR wavelengths under similar conditions. Surface LID thresholds were determined for the three distinct stages of damage at four MIR wavelengths. These studies deliver significant insights into the involved damage mechanisms at the MIR wavelengths.

Ultrafast melting, followed by amorphization, was observed, like in NIR experiments, but it occurs well above the plasma critical density, despite the prediction of the high-collision-rate Drude model. Spallative ablation of a few layers of silicon is the only means by which oxide layer removal was observed with MIR wavelengths. The nanoroughness of the remaining material after ablation decreases with wavelength.

Qualitative analysis of the absorption mechanisms and associated electron excitations by the ultrashort laser pulses suggests attributing the maximum of the wavelength scaling of LID thresholds to simultaneous action of several effects. They include a transition from domination of indirect inter-band excitation to domination of direct inter-band excitation; laser-driven modification of energy bands; enhancement of tunneling electron emission; and the transition from photon-driven absorption regime the field-driven regime. As discussed above, the shorter (e. g., NIR) wavelengths are favorable for dominating contributions of the multiphoton absorption to the inter-band electron promotion, intra-band excitation, and electron emission. In contrast, increase of laser wavelength towards longer-wavelength part of the MIR range leads to reduction of photon energy, increase of ablation thresholds, and corresponding increase of ponderomotive energy of the oscillating conduction electrons. Respectively, the probability of all the multiphoton effects reduces, but the contributions of the field effects increases with increase of laser wavelength. That transition between the absorption regimes is associated with increase of the contribution of ponderomotive energy to the intra-band electron excitation and overall absorption, resulting a decreased damage threshold. Therefore, the wavelength scaling of the melting and ablation thresholds may reflect a transition from the photon-dominated regime of absorption to the field-dominated regime. More detailed justification of this hypothesis requires extended ab initio simulations not available currently.

## Methods

### LID experiments

The laser source was The Ohio State University (OSU) Extreme Mid-Infrared (EMIR) optical parametric amplifier (OPA)^1,70,71^. A schematic of the experiment is shown in Fig. S1. 1-on-1 LID experiments were performed in air, with p-polarized pulses (*λ* = 2.75, 3.15, 3.75, 4.15 μm, ${\tau }_{{\rm{FWHM}}}=200\,{\rm{fs}}$) and an angle of incidence of 31 degrees. By varying the pulse energy, the peak fluence was varied from 0.25 to 2 Jcm^−2^. The energy of every pulse was recorded. The beam profile of the focal spot illuminating the surface of the sample was recorded once for each wavelength, and focal spot sizes ranged from $20-25$ μm FWHM (geometric mean of the horizontal and vertical spot sizes). Reported fluences are calculated after projection of the measured focal spot profile onto the sample surface (surface normal fluence). The sample used was an *n*-type single crystal Si (100)/<110> (${\rm{resistivity}}\,{ > 10}^{3}$Ω-cm with intrinsic carrier concentration ∼10^14^ cm^−3^); electric field polarization was primarily in the (1 1 1) direction. *Ex-situ* SEM imaging was performed on each damage site. AFM imaging was performed on a subset of damage sites. Cross sectional TEM and spatially-resolved *μ*-Raman spectroscopy were both performed on a single damage site.

### The two traditional simulation approaches

Keldysh simulations were performed using Python simulation code^72^. This code uses the Keldysh equations described in the supplemental materials. The minimum peak incident laser intensity required for ultrafast melting, i.e. for photoionization of 7% of the valence band electrons, was determined to be the LIDT. Once the conduction band electron density just barely reaches the threshold at the surface, ultrafast melting of the surface layer occurs. In this work, our simulations consider direct transitions from the valence band $\Gamma$ valley to the conduction band with the positive effective mass. This is indicated through transition number 2 in Fig. 6a. Specific material parameter inputs for silicon are summarized in Supplemental Materials. Additional details are provided in the supplemental materials.

### Estimations of the probabilities of direct and indirect transitions

For derivation of the estimation of Eq. (4) of the main text, we assume that the virtual states V1 and V2 are reached via a direct transition by absorption of several laser photons. Correspondingly, the energy gap between the Γ point of the valence bands and the virtual states is evaluated as follows:

6 $$\begin{array}{c}{E}_{{\rm{V}}1}=\langle \frac{{\Delta }_{{\rm{INDGV}}1}}{\hslash \omega }+1\rangle ,\,{E}_{{\rm{V}}2}=\langle \frac{{\Delta }_{{\rm{INDGV}}2}}{\hslash \omega }+1\rangle ,\end{array}$$

where Δ_INDGv2_ = 1.12 eV for the Γ-to-X transition (5 in Fig. 6a), and Δ_INDGv1_ = 2.00 eV for the Γ-to-L transition (4 in Fig. 6a)^34^. Probability of the electron transitions from the top Γ-states of the valence bands to the virtual states per time interval *δt* estimated by the uncertainty relations of Eq. (3) can be evaluated via the multiphoton-ionization rate *W*_*V*1_ and *W*_*V2*_:

7 $$\begin{array}{c}{P}_{{\rm{V}}1}=\frac{{W}_{{\rm{V}}1}\delta {t}_{{\rm{V}}1}}{{N}_{{\rm{VB}}}},\,{P}_{{\rm{V}}2}=\frac{{W}_{{\rm{V}}2}\delta {t}_{{\rm{V}}2}}{{N}_{{\rm{VB}}}}\end{array}$$

where *N*_VB_ is the total population of the involved valence bands. The other process involved into the indirect transitions 4 and 5 (Fig. 6) considers electron-phonon collisions^12,19^. Probability *P*_elph_ of this collision process per time interval *δt* can be evaluated as follows^12,19^:

8 $$\begin{array}{c}{P}_{{\rm{eph}}}={\Gamma }_{{\rm{eph}}}\delta t=\frac{\delta t}{{\tau }_{{\rm{eph}}}}\end{array}$$

where *Γ*_eph_ = 1/*τ*_eph_ is the rate of electron collisions with polar phonons^12,19,34^. The total probability of the transitions 4 and 5 can be evaluated in the first approximation as a product of the probabilities of Eqs. (7) and (8) by assuming the electron excitation to the virtual states and the electron-phonon collisions are independent:

9 $$\begin{array}{c}{P}_{4}={P}_{{\rm{V}}1}{P}_{{\rm{eph}}}=\frac{{W}_{{\rm{V}}1}\delta {t}_{{\rm{V1}}}^{2}}{{N}_{{\rm{VB}}}{\tau }_{{\rm{eph}}}},\,{P}_{5}={P}_{{\rm{V}}2}{P}_{{\rm{eph}}}=\frac{{W}_{{\rm{V}}2}\delta {t}_{{\rm{V2}}}^{2}}{{N}_{{\rm{VB}}}{\tau }_{{\rm{eph}}}}.\end{array}$$

In Eq. (8), the rate of the electron-phonon collisions considers all the collisions that result in variations of electron momentum, i.e., all contributions to the momentum transfer from phonons to the excited electrons of the virtual states. Characteristic time *τ*_eph_ of the electron-phonon momentum transfer (also referred to as momentum dephasing time) varies from few tens to few hundreds of femtoseconds in typical semiconductors^50–52^ depending on laser and material parameters.

To obtain the estimation of Eq. (4), the probability of the direct Γ-point transitions is evaluated via the inter-band photoionization rate *W*_MP2_ as follows:

10 $$\begin{array}{c}{P}_{2}=\frac{{W}_{MP2}\delta {t}_{i}}{{N}_{VB}},\,i=V1,\,V2.\end{array}$$

The photoionization rates are estimated using the Keldysh formula^16^ with corresponding corrections.

## Supplementary information

Supplementary Information

## Data Availability

The data that support the findings of this study are available from the corresponding author upon reasonable request.

## References

[1] Shcherbakov MR (2019). Photon acceleration and tunable broadband harmonics generation in nonlinear time-dependent metasurfaces. Nat. Commun..

[2] Li J (2017). 53-attosecond X-ray pulses reach the carbon K-edge. Nature Communications.

[3] Tochitsky S (2019). Megafilament in air formed by self-guided terawatt long-wavelength infrared laser. Nature Photonics.

[4] Baudisch M (2018). Ultrafast nonlinear optical response of Dirac fermions in graphene. Nat. Commun..

[5] Silva REF, Blinov IV, Rubtsov AN, Smirnova O, Ivanov M (2018). High-harmonic spectroscopy of ultrafast many-body dynamics in strongly correlated systems. Nature Photonics.

[6] Nejadmalayeri A (2005). Inscription of optical waveguides in crystalline silicon by mid-infrared femtosecond laser pulses. Opt. Lett..

[7] Thire N (2018). Highly stable, 15 W, few-cycle, 65 mrad CEP-noise mid-IR OPCPA for statistical physics. Optics Express.

[8] Pogorelsky IV, Babzien M, Ben-Zvi I, Skaritka J, Polyanskiy MN (2016). BESTIA - The next generation ultra-fast CO_2_ laser for advanced accelerator research. Nuclear Instruments & Methods in Physics Research Section A-Accelerators Spectrometers Detectors and Associated Equipment.

[9] Pires H, Baudisch M, Sanchez D, Hemmer M, Biegert J (2015). Ultrashort pulse generation in the mid-IR. Prog. Quantum Electron..

[10] Austin DR (2018). Femtosecond laser damage of germanium from near- to mid-infrared wavelengths. Opt. Lett..

[11] Simanovskii D, Schwettman H, Lee H, Welch A (2003). Midinfrared optical breakdown in transparent dielectrics. Phys. Rev. Lett..

[12] Bassani F, Parravicini GP, Ballinger RA, Birman JL (1976). Electronic States and Optical Transitions in Solids. Physics Today.

[13] Guizard S (1996). Time-resolved studies of carriers dynamics in wide band gap materials. Nuclear Instruments & Methods in Physics Research Section B-Beam Interactions with Materials and Atoms.

[14] Martin P (1997). Subpicosecond study of carrier trapping dynamics in wide-band-gap crystals. Physical Review B.

[15] Kaiser A, Rethfeld B, Vicanek M, Simon G (2000). Microscopic processes in dielectrics under irradiation by subpicosecond laser pulses. Physical Review B.

[16] Keldysh L (1965). Ionization in Field of a Strong Electromagnetic Wave. Soviet Physics Jetp-Ussr.

[17] Wood, R. M. In *Laser-induced damage of optical materials* (Institute of Physics, Bristol, 2003).

[18] Gallais L (2015). Wavelength dependence of femtosecond laser-induced damage threshold of optical materials. J. Appl. Phys..

[19] Ashcroft, N. W. & Mermin, N. D. In *Solid state physics* (Holt, Rinehart and Winston, New York, 1976).

[20] Landau, L. D. & Lifshits, E. M. In *Quantum mechanics*, *non-relativistic theory*, (1958).

[21] Anderson, B. L. & Anderson, R. L. In *Fundamental mechanisms of ultrafast laser-induced band-structure modification in non-metal crystals*. (McGraw-Hill Higher Education, New York, 2004).

[22] Bonse J, Baudach S, Kruger J, Kautek W, Lenzner M (2002). Femtosecond laser ablation of silicon-modification thresholds and morphology. Appl. Phys. A-Mater. Sci. Process..

[23] Shaheen ME, Gagnon JE, Fryer BJ (2014). Femtosecond laser ablation behavior of gold, crystalline silicon, and fused silica: a comparative study. Laser Phys..

[24] Zayarny DA (2016). Surface ablation of aluminum and silicon by ultrashort laser pulses of variable width. Jetp Lett..

[25] Kafka, K. R. P. Laser-induced damage with femtosecond pulses. (The Ohio State University, 2017).

[26] Rethfeld B, Ivanov DS, Garcia ME, Anisimov SI (2017). Modelling ultrafast laser ablation. Journal of Physics D-Applied Physics.

[27] Allenspacher P, Huttner B, Riede W (2002). Ultrashort pulse damage of Si and Ge semiconductors. Laser-Induced Damage in Optical Materials.

[28] Austin D, Kafka K, Blaga CI, Dimauro LF, Chowdhury EA (2014). Measurement of femtosecond laser damage thresholds at mid IR wavelengths. Laser-Induced Damage in Optical Materials.

[29] Gamaly E, Rode A, Luther-Davies B, Tikhonchuk V (2002). Ablation of solids by femtosecond lasers: Ablation mechanism and ablation thresholds for metals and dielectrics. Phys Plasmas.

[30] Agranat MB (2006). On the mechanism of the absorption of femtosecond laser pulses in the melting and ablation of Si and GaAs. Jetp Lett..

[31] Pronko P (1998). Avalanche ionization and dielectric breakdown in silicon with ultrafast laser pulses. Phys. Rev. B.

[32] Balling P, Schou J (2013). Femtosecond-laser ablation dynamics of dielectrics: basics and applications for thin films. Rep. Prog. Phys..

[33] Starke K (2003). Investigations in the nonlinear behavior of dielectrics by using ultrashort pulses. Laser-Induced Damage in Optical Materials: 2003.

[34] Adachi, S. In *Properties of Group-IV*, *III-V and II-VI Semiconductors* (Blackwell Science Publ., Oxford, 2005).

[35] Bonse J, Brzezinka K, Meixner A (2004). Modifying single-crystalline silicon by femtosecond laser pulses: an analysis by micro Raman spectroscopy, scanning laser microscopy and atomic force microscopy. Appl. Surf. Sci..

[36] von der Linde D, Sokolowski-Tinten K, Bialkowski J (1997). Laser-solid interaction in the femtosecond time regime. Appl. Surf. Sci..

[37] Sokolowski-Tinten K, von der Linde D (2000). Generation of dense electron-hole plasmas in silicon. Physical Review B.

[38] Stampfli P, Bennemann K (1990). Theory for the Instability of the Diamond Structure of Si, Ge, and C Induced by a Dense Electron-Hole Plasma. Phys. Rev. B.

[39] Stampfli P, Bennemann K (1992). Dynamic Theory of the Laser-Induced Lattice Instability of Silicon. Physical Review B.

[40] Stampfli P, Bennemann K (1994). Time-Dependence of the Laser-Induced Femtosecond Lattice Instability of Si and Gaas - Role of Longitudinal Optical Distortions. Physical Review B.

[41] Liu J (1982). Simple Technique for Measurements of Pulsed Gaussian-Beam Spot Sizes. Opt. Lett..

[42] Cullis A, Webber H, Chew N, Poate J, Baeri P (1982). Transitions to Defective Crystal and the Amorphous State Induced in Elemental Si by Laser Quenching. Phys. Rev. Lett..

[43] Yater J, Thompson M (1989). Orientation Dependence of Laser Amorphization of Crystal Si. Phys. Rev. Lett..

[44] Otobe T, Shinohara Y, Sato SA, Yabana K (2016). Femtosecond time-resolved dynamical Franz-Keldysh effect. Physical Review B.

[45] Private communication with K. Yabana.

[46] Stuart B (1996). Nanosecond-to-femtosecond laser-induced breakdown in dielectrics. Physical Review B.

[47] Shugaev MV (2016). Fundamentals of ultrafast laser-material interaction. MRS Bull.

[48] https://www.crystran.co.uk/optical-materials/silica-glass-sio2.

[49] https://escooptics.com/blogs/news/the-benefits-of-fused-silica-quartz.

[50] Hu B (1995). Identifying the Distinct Phases of Carrier Transport in Semiconductors with 10-fs Resolution. Phys. Rev. Lett..

[51] Kash J, Tsang J, Hvam J (1985). Subpicosecond Time-Resolved Raman-Spectroscopy of LO Phonons in GaAs. Phys. Rev. Lett..

[52] Sjakste J, Tanimura K, Barbarino G, Perfetti L, Vast N (2018). Hot electron relaxation dynamics in semiconductors: assessing the strength of the electron-phonon coupling from the theoretical and experimental viewpoints. J. Phys.: Condens. Matter.

[53] Dachraoui H, Husinsky W, Betz G (2006). Ultra-short laser ablation of metals and semiconductors: evidence of ultra-fast Coulomb explosion. Applied Physics A-Materials Science & Processing.

[54] Gruzdev V, Komolov V (2004). The action of ultrashort laser pulses on a semiconductor: Possible processes and a possible sequence of events. Journal of Optical Technology.

[55] Anisimov S, Benderskii V, Farkas G (1977). Nonlinear Photoelectric Effect in Metals Produced by a Laser-Radiation. Uspekhi Fiz. Nauk.

[56] Gamaly EG, Rode AV (2018). Ultrafast re-structuring of the electronic landscape of transparent dielectrics: new material states (Die-Met). Appl. Phys. A.

[57] Hsu W-H (2018). Enhancement of X-ray emisson from nanocolloidal gold suspension under double-pulse excitation. Beilstein J. Nanotechnol..

[58] Sokolowski-Tinten K, Cavalleri A, von der Linde D (1999). Single-pulse time- and fluence-resolved optical measurements at femtosecond excited surfaces. Applied Physics A-Materials Science & Processing.

[59] Sergaeva O, Gruzdev V, Austin D, Chowdhury E (2018). Ultrafast excitation of conduction-band electrons by high-intensity ultrashort laser pulses in band-gap solids: Vinogradov equation versus Drude model. Journal of the Optical Society of America B-Optical Physics.

[60] Gruzdev, V., Austin, D. R., Sergaeva, O. N. & Chowdhury, E. *Beyond the Drude Approach: a Keldysh-Vinogradov Model of Dynamics of Ultrafast Laser-Induced Electron Excitation*, Optical Society of America, San Jose, California, 2017).

[61] Gruzdev VE (2007). Photoionization rate in wide band-gap crystals. Physical Review B.

[62] Yacoby Y (1968). High-Frequency Franz-Keldysh Effect. Physical Review.

[63] Keldysh L (1958). The Effect of a Strong Electric Field on the Optical Properties of Insulating. Crystals. Soviet Physics Jetp-Ussr.

[64] Mizumoto Y, Kayanuma Y (2005). Band renormalization of semiconductors by high-intensity infrared laser: One-dimensional model. Physical Review B.

[65] Mizumoto Y, Kayanuma Y, Srivastava A, Kono J, Chin AH (2006). Dressed-band theory for semiconductors in a high-intensity infrared laser field. Physical Review B.

[66] Prabhu S, Vengurlekar A (2004). Dynamics of the pump-probe reflectivity spectra in GaAs and GaN. J. Appl. Phys..

[67] Bennett B, Soref R, Delalamo J (1990). Carrier-Induced Change in Refractive-Index of InP, GaAs, and InGaAsP. IEEE J. Quant. Electron..

[68] Lee Y (1986). Room-Temperature Optical Nonlinearities in Gaas. Phys. Rev. Lett..

[69] Gruzdev V (2004). Analysis of the transparent-crystal ionization model developed by L. V. Keldysh. Journal of Optical Technology.

[70] Werner, K. Ultrafast mid-infrared laser-solid interactions. (The Ohio State University, 2019).

[71] Werner K (2019). Ultrafast mid-infrared high harmonic and supercontinuum generation with n_2_ characterization in zinc selenide. Optics Express.

[72] Austin, D. R. Semiconductor surface modification using mid-infrared, femtosecond laser pulses. (The Ohio State University, 2017).

