# Supplementary material for: Single-Shot Multi-Stage Damage and Ablation of Silicon by Femtosecond Mid-infrared Laser Pulses
Source: Sci Rep. 2019 Dec 27;9:19993. doi: 10.1038/s41598-019-56384-0 (PMC6934619; doi:10.1038/s41598-019-56384-0)
Supplement: Supplementary file 1 — Supplementary Information [file 41598_2019_56384_MOESM1_ESM.pdf]

SUPPLEMENTARY MATERIALS

Single-Shot Multi-Stage Damage and Ablation of Silicon by Femtosecond Mid-infrared Laser Pulses

Kevin Werner, Vitaly Gruzdev, Noah Talisa, Kyle Kafka, Drake Austin, Carl M. Liebig, and Enam Chowdhury

This PDF file includes:

1. **Setup: Laser and spectroscopy setup and parameters: 2**

Fig. S1. 1-on-1 setup for laser-induced damage experiments.

2. **Extra images of damage spots: 3**

Fig. S2. Representative damage spots for 3 different wavelengths at relatively high fluences.
Fig. S3. Side-by-side SEM images of a 2.75 and 4.15 um damage site at the same fluence (0.85 J/cm^2).
Fig. S4. Focused ion beam milling sample preparation for cross-sectional TEM.

3. **Band-structure parameters at the Γ, L, and X points of the Brillouin zone: 5**

Table S1: Effective electron masses and energy gaps at Γ, L, and X points of the Brillouin zone.
Table S2. Effective hole masses (in units of free electron mass) at the Γ point of Brillouin zone.

4. **Evaluation of the photoionization rates with the Keldysh formula: 6**

Supplementary text.
Fig. S5. Intensity scaling of the photoionization rate at the Γ point, crystal direction <111>.
Fig. S6. Intensity scaling of the photoionization rate for the direct inter-band transitions at the Γ, L, and X points.
Fig. S7: Intensity scaling of the transitions rates from the light-hole valence band to the Γ point of conduction band and to the virtual states V1 and V2 (at free-electron effective mass)
Fig. S8: Intensity scaling of the transitions rates from the light-hole valence band to the Γ point of conduction band and to the virtual states V1 and V2 (at LH-valence-band and conduction-band values of effective mass)

5. **Time-domain simulations with the Keldysh formula: 16**

Supplementary text.
Fig. S9: Results of time-domain simulation with the Keldysh formula.
Fig. S10: Keldysh photoionization simulation with multiple valence bands and valance band depletion.

6. **Simulations with the Gamaly model: 19**

Supplementary text.

7. **Microscopic criteria of damage/ablation threshold in semiconductors: 20**

Supplementary text.

1. 1-on-1 setup for laser-induced damage experiments

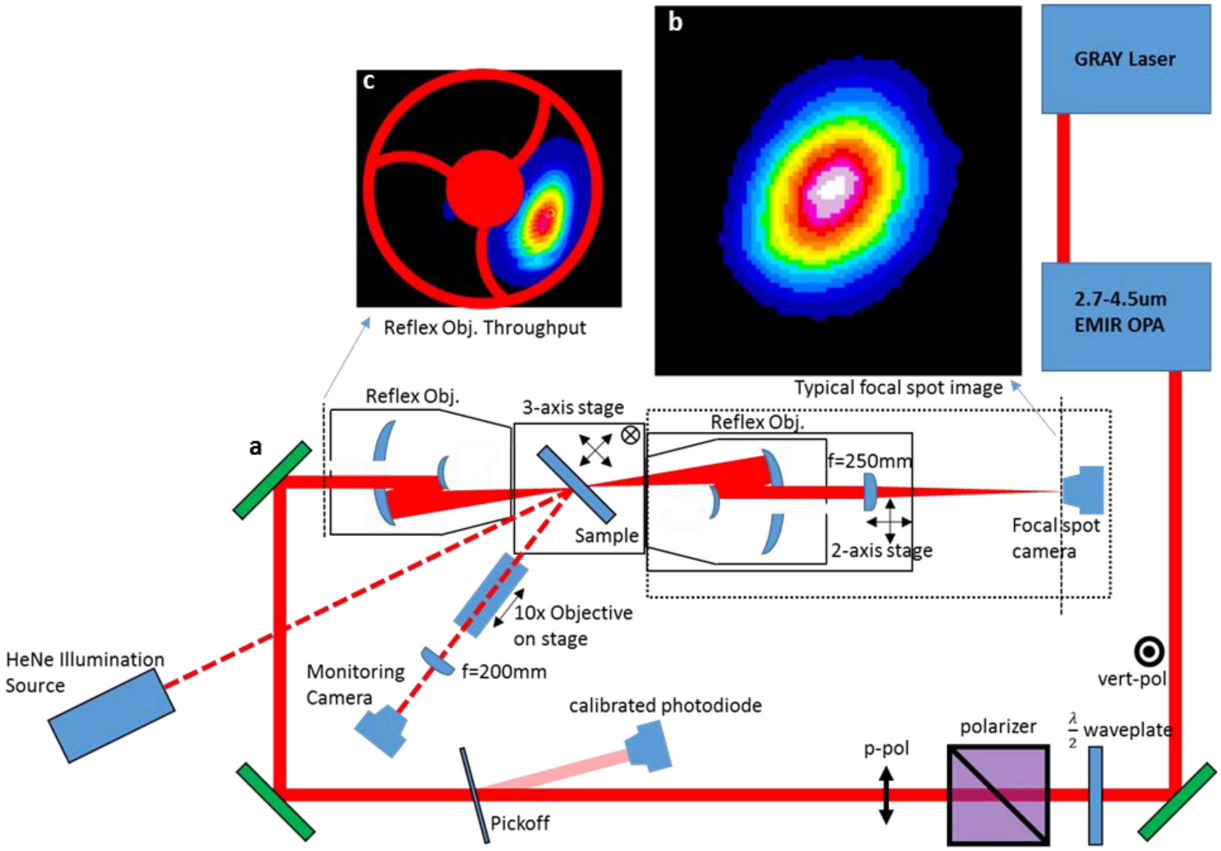

Figure S1: 1-on-1 setup for laser-induced damage experiments. (a) τ_{FWHM}=200 fs pulses from EMIR OPA (λ=2.75,3.15,3.75,4.15 μm) were sent through a $\lambda/2$ waveplate / horizontal polarizer for pulse energy control. A low-energy pickoff illuminates a calibrated photodiode to record the pulse energy of each shot. P-polarized pulses are focused down to a spot size 20-25 um at a 31 degrees angle of incidence (using an infinite conjugate, gold coated, 15X/0.28NA HP reflex objective). A HeNe laser illuminates the sample for in-situ imaging. A second reflex objective combined with a 250mm PLCX lens is used to image the focus before and after each run. A fiducial placed in the sample plane was imaged on both the in-situ and focal spot imaging systems to ensure the part of the focus being imaged was the same part illuminating the sample surface. (b) Typical focal spot image ($\lambda = 2.7um$). (c) Throughput of the reflex objective. An artificial overlay shows how the beam is aligned to go through one of the three areas on the side of the objective for maximum energy throughput.

2. Extra images of damage spots

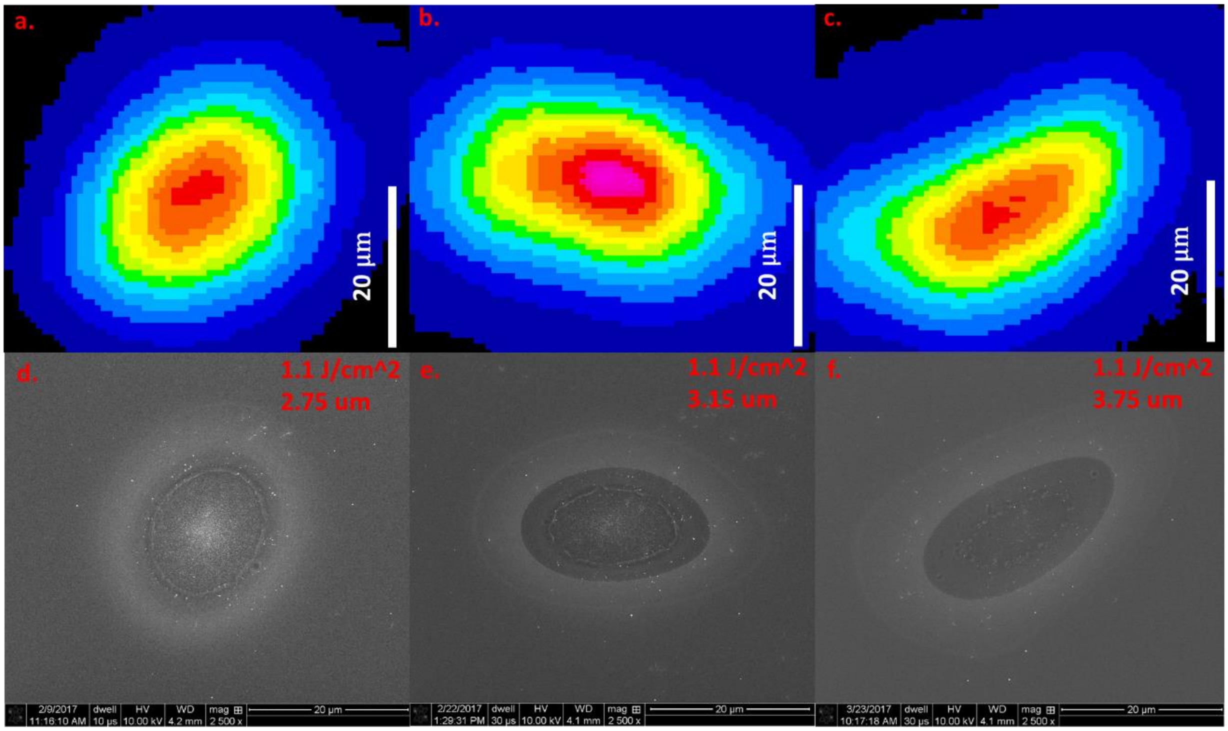

Figure S2: Representative damage spots for 3 different wavelengths at relatively high fluences. (a)-(c) are measured focal spot images taken for each of the damage sites (directly above their corresponding damage sites). (d)-(f) show damage sites at 3 different wavelengths 2.75 um (d), 315 um (e), and 3.75 um (f) for a fixed fluence of 1.1 J/cm^2.

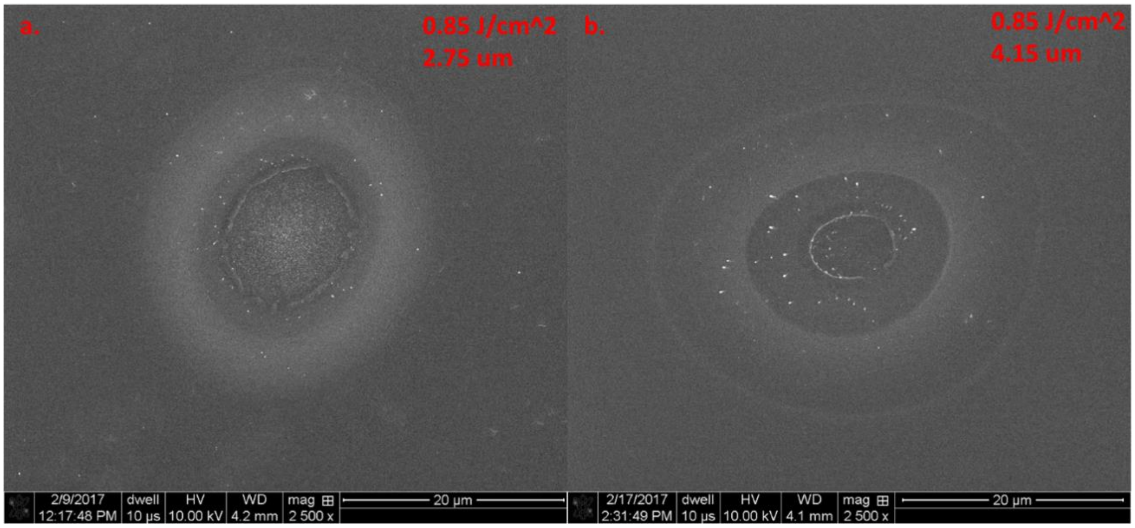

Figure S3: Side-by-side SEM images of a 2.75 um (a) and 4.15 um (b) damage site at the same fluence (0.85 J/cm^2).

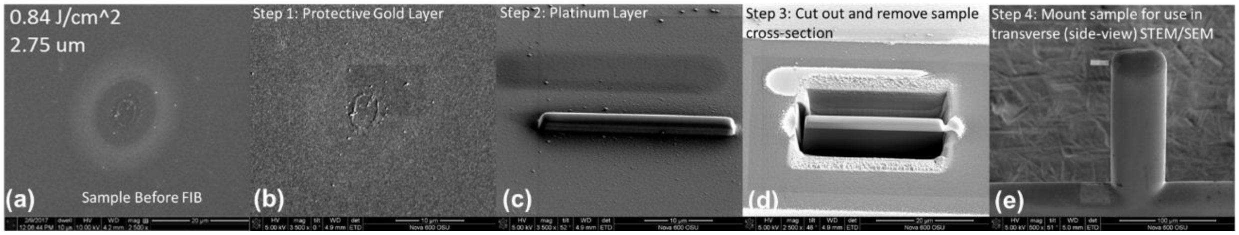

Figure S4: Focused ion beam milling sample preparation for cross-sectional TEM. (a) The sample, which underwent TEM, is the same exact sample from Fig. S3 (a) (0.84 J/cm^2 fluence at λ=2.75μm). (b) First, a 50 nm protective gold is deposited to prevent charging. (c) Then, a sacrificial platinum layer is deposited to protect the underlying sample. (d) Cadmium ions bombard the sample digging three trenches. A nanomanipulator is attached to the cross section (with further Pt deposition) before the final fourth trench is dug, detaching the cross section from the sample. (e) The sample is lifted out and mounted for cross sectional TEM. The sample is thinned in sections until it is transparent to electrons (~50 nm) using more gallium ions. FIB preparation was performed at the Center for Electron Microscopy and Analysis (CEMAS) at The Ohio State University, Columbus, OH.

3. Band-structure parameters at the Γ, L, and X points of the Brillouin zone

The relations employed for evaluation of the inter-band transition rates, intra-band absorption, and impact-ionization rate contain effective electron masses and direct energy gaps between involved valence and conduction bands. Since the transitions are expected to happen at Γ, L, and X points of the Brillouin zone, relevant parameters are summarized in the following two tables.

Table S1: Effective electron masses and energy gaps at Γ, L, and X points of the Brillouin zone. The energy gaps are shown between the lowest conduction band and the highest valence band at temperature 300 K as they are available from Refs. [3, 4].

Parameter	Γ point	X valley	L valley
Direct energy gap [eV]	From 3.28 to 3.5 eV	4.27 eV	3.30 eV
Transverse effective electron mass (units of free-electron mass)	0.188	0.1905	0.130
Longitudinal effective electron mass (unites of free-electron mass)	0.188	0.916 (direction <100>)	1.418 (direction <111>)
Effective hole mass	See Table S2	-1.2[a]	-1.6[a]

[a] Estimations based on the graphical representations of the silicon band structure of Refs. [3, 4].

Table S2. Effective hole masses (in units of free electron mass) at the Γ point of Brillouin zone. The data are summarized for the band structure of crystalline silicon based on the data of Refs. [3, 4].

Hole effective mass	Direction <100>	Direction <111>
Light hole valence band	0.202	0.139
Heavy-hole valence band	0.277	0.718
Split-off valence band	0.290	0.290

4. Evaluation of the photoionization rates with the Keldysh formula

To evaluate the photoionization rate of the direct inter-band electron transitions around the Γ point of the first Brillouin zone, energy-momentum relations are approximated by the Kane relation for the heavy-hole (HH) and light-hole (LH) valence bands [1-4]:

$$\varepsilon_K(\vec{p}) = \Delta\sqrt{1 + \frac{p^2}{m_i\Delta}}, \quad \text{(S1)}$$

where Δ is the direct band gap at the Γ point, *m* is reduced effective electron-hole mass evaluated from the electron m_{CB} and hole m_{iH} effective masses as follows [2]:

$$\frac{1}{m_i} = \frac{1}{m_{iH}} + \frac{1}{m_{CB}}. \quad \text{(S2)}$$

Here *i* = L for light-hole (LH) and *i* = H for heavy-hole (HH) valence band. For the split-off (SO) valence band, a parabolic energy-momentum relation is employed [3, 4]:

$$\varepsilon_P(\vec{p}) = \Delta\left(1 + \frac{p^2}{2m_i\Delta}\right), \quad \text{(S3)}$$

The conduction band (CB) is described by a parabolic energy-momentum relation [2-4].

For the direct electron transitions from the HH and LH valence bands, this approximation allows use of the Keldysh formula for the photoionization rate [1]. After fixing some minor misprints in the original Keldysh formula of Ref. [1], we use the following relations to evaluate the rates of LH-to-CB and HH-to-CB direct inter-band transitions [5]:

$$w_{NP} = 2N\cdot\frac{2\omega}{9\pi}\cdot\left(\frac{\sqrt{1+\gamma^2}}{\gamma}\cdot\frac{m\omega}{\hbar}\right)^{3/2}\cdot Q_{NP}\left(\gamma, \frac{\tilde{\Delta}_{NP}}{\hbar\omega}\right)\cdot\exp\left[-\pi\left\langle\frac{\tilde{\Delta}_{NP}}{\hbar\omega}+1\right\rangle\cdot\frac{K(\phi)-E(\phi)}{E(\theta)}\right], \quad \text{(S4)}$$

where the factor *N* is introduced to account for degeneracy of the conduction band. We assumed *N*=2 for the transitions from each valence band to the double-degenerated lowest conduction band at the Γ point. For the Γ-point transitions, the effective mass of one of the conduction-band branches is negative according to the E-k diagram of silicon [3, 4]. However, a reliable estimation of its value is not available in publications. Due to this reason, the contribution of that conduction band is accounted for by introduction of the factor *N* above. This approach delivers only order-of-magnitude estimation of the direct inter-band transition rates, but this accuracy is enough to support the qualitative discussions of the main text. The slowly-varying amplitude is introduced as follows [1, 5]:

$$Q_{NP}(\gamma, x) = \sqrt{\frac{\pi}{2\cdot K(\theta)}}\cdot\sum_{n=0}^{\infty}\exp\left\{-\pi\cdot\frac{K(\phi)-E(\phi)}{E(\theta)}\cdot n\right\}\cdot\Phi\left\{\sqrt{\frac{\pi^2(\langle x+1\rangle - x + n)}{2\cdot K(\theta)\cdot E(\theta)}}\right\}, \quad \text{(S5)}$$

where $\theta = \frac{1}{\sqrt{1+\gamma^2}}, \phi = \frac{\gamma}{\sqrt{1+\gamma^2}}$ are the arguments of the complete elliptic integrals *K* and *E*; Φ is Dawson integral:

$$\Phi(x) = \int_0^x \exp(\xi^2 - x^2)\,d\xi, \quad \text{(S6)}$$

effective band gap reads as follows:

$$\tilde{\Delta}_{NP} = \frac{2}{\pi}\Delta\cdot\left[\frac{\sqrt{1+\gamma^2}}{\gamma}\cdot E\left(\frac{1}{\sqrt{1+\gamma^2}}\right)\right], \quad \text{(S7)}$$

and the Keldysh adiabatic parameter keeps the original definition of Ref. [1]:

$$\gamma = \frac{\omega\sqrt{m\Delta}}{eF}. \tag{S8}$$

For the parabolic relation of Eq. (S3), the formula of Ref. [6] with corrected pre-exponential factor of Ref. [7] is utilized to evaluate the photoionization rate:

$$w = 2\frac{\omega}{8\pi}\left(\frac{m\omega}{\hbar}\right)^{3/2}\exp\left\{-2\left\langle\frac{\tilde{\Delta}}{\hbar\omega}+1\right\rangle\left(\sinh^{-1}\left(\gamma\sqrt{2}\right)-\frac{\gamma\sqrt{2}}{\sqrt{1+2\gamma^2}}\right)-2\frac{\tilde{\Delta}}{\hbar\omega}\frac{2\sqrt{2}\gamma^3}{\sqrt{1+2\gamma^2}\left(1+4\gamma^2\right)}\right\}Q_p\left(\gamma,\frac{\tilde{\Delta}}{\hbar\omega}\right),$$

(S9)

where the slowly-varying amplitude reads as follows:

$$Q_p(\gamma,x)=\sqrt{\frac{\sqrt{1+2\gamma^2}}{\gamma\sqrt{2}}}\sum_{n=0}^{\infty}\exp\left\{-2n\left(\sinh^{-1}\left(\gamma\sqrt{2}\right)-\frac{\gamma\sqrt{2}}{\sqrt{1+2\gamma^2}}\right)\right\}\Phi\left\{\sqrt{2\left(n+\langle x+1\rangle-x\right)\frac{\gamma\sqrt{2}}{\sqrt{1+2\gamma^2}}}\right\}$$

, (S10)

and effective band gap is evaluated as follows:

$$\tilde{\Delta}_P = \Delta\cdot\left(1+\frac{1}{4\gamma^2}\right)=\Delta\cdot\left(1+\frac{e^2\cdot F^2}{4\cdot m\cdot\Delta\cdot\omega^2}\right). \tag{S11}$$

The Dawson function and the adiabatic parameter are given by Eqs. (S6) and (S8) respectively.

For the L-point photoionization transitions, we bear in mind the fact that the effective mass of the valence band is negative [3, 4], i. e., the reduced effective electron-hole mass of the L-point transitions is defined via the L-valley longitudinal electron effective mass m_{eLl} and the valence-band effective hole mass m_{hL} the as follows:

$$\frac{1}{m}=\frac{1}{m_{eLl}}-\frac{1}{m_{hL}}. \tag{S12}$$

Estimation of the valence-band effective mass of the L point is about one free-electron mass [3, 4]. The parabolic energy-momentum relation of Eq. (S3) is utilized, and the photoionization rate is evaluated by Eqs. (S9) – (S11) around the L point of the Brillouin zone.

Intensity scaling of the photoionization rate at the Γ point, crystal direction <111>

Figs. 6B of main text represent the intensity scaling of the rate of inter-band photoionization transitions at the Γ point at 4150 nm. Three figures below depict the intensity scaling of the rates of the Γ-point inter-band transitions at wavelengths 2750, 3150, and 3750 nm.

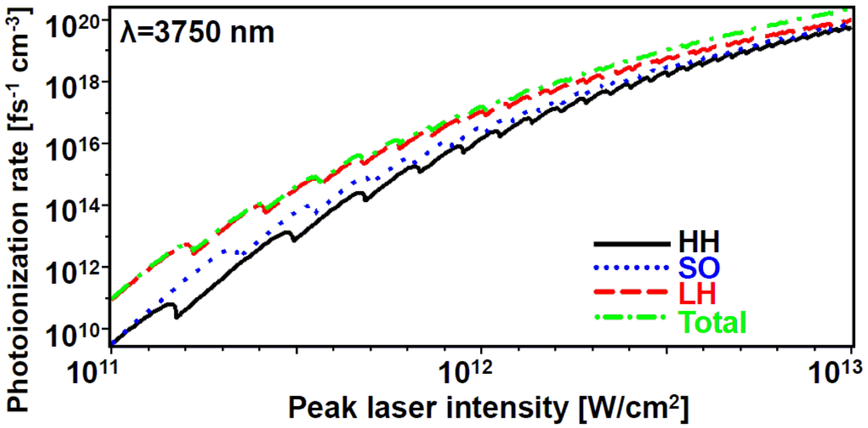

A

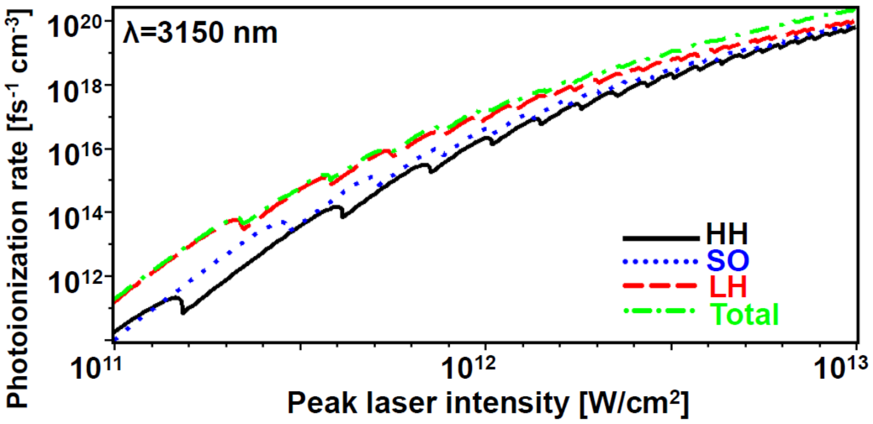

B

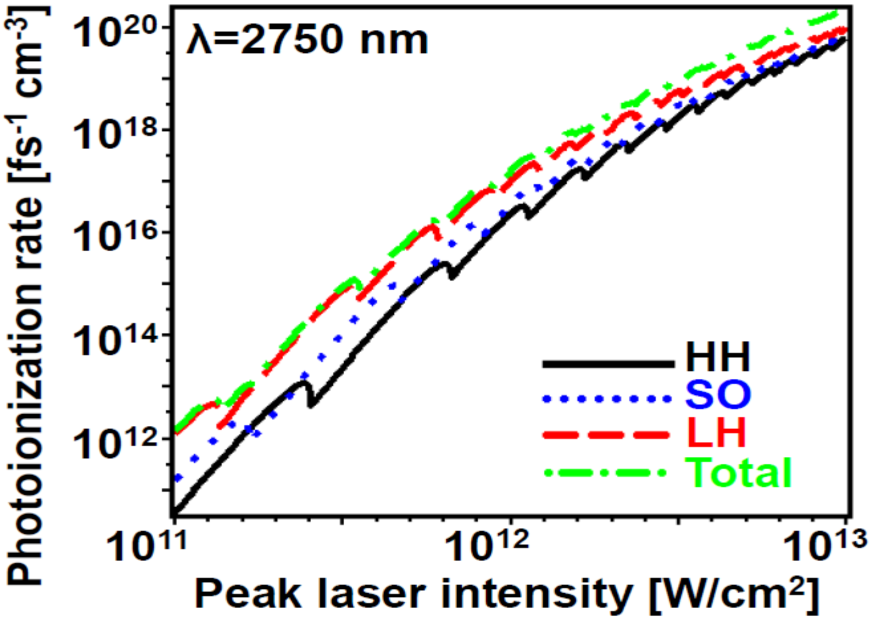

C

Figure S5: Intensity scaling of the photoionization rate_at the Γ point, crystal direction <111>. Intensity scaling of the photoionization rate estimated at 3750 nm (A), 3150 nm (B) and 2750 nm (C) by Eqs. (S1) through (S12) for the parameters of the three valence bands and two conduction bands at the Γ point, crystal direction <111>. Green dash-dotted line – the total rate of the electron transitions from all the three valence bands; red dashed line – the rate for the transitions from LH band; blue dotted line – from HH valence band; black solid line – from SO band.

Intensity scaling of the photoionization rate for the direct inter-band transitions at the Γ, L, and X points

The photoionization rate is evaluated at Γ, L, and X points of the Brillouin zone (crystal direction <111>) at all the four wavelengths utilized in the experiments. For the Γ point, only contribution of the light-hole valence band and the total rate for the three valence bands are shown.

The most remarkable feature of all the simulations is that the rate of direct inter-band transitions to the L valley is several orders of magnitude lower than those of the transitions around Γ and X points. This is explained by the specific structure of the energy bands around the L point of the Brillouin zone that supports the low-rate multiphoton regime of the photoionization (the values of the adiabatic Keldysh parameter are between 4.0 and 7.5 for the L-point transitions at the threshold intensities for all the tested wavelengths).

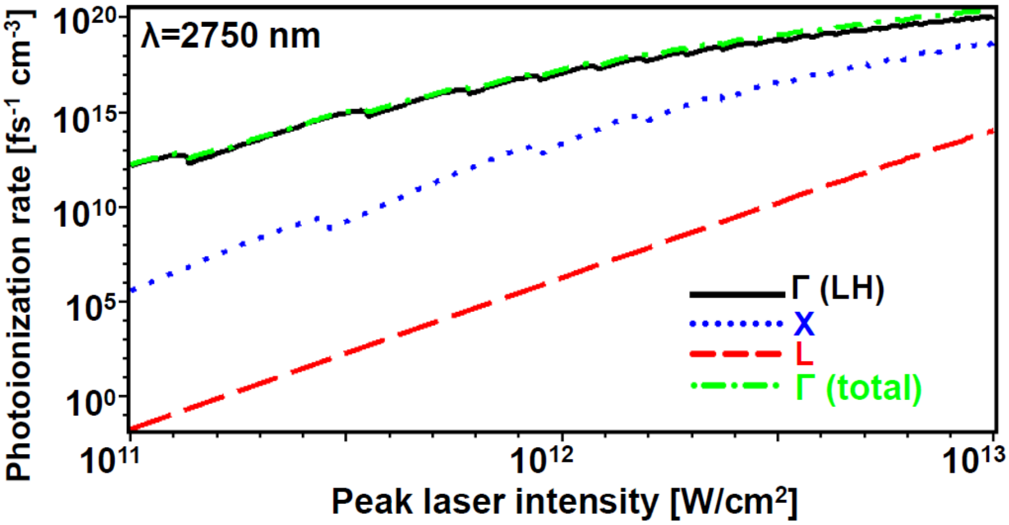

A

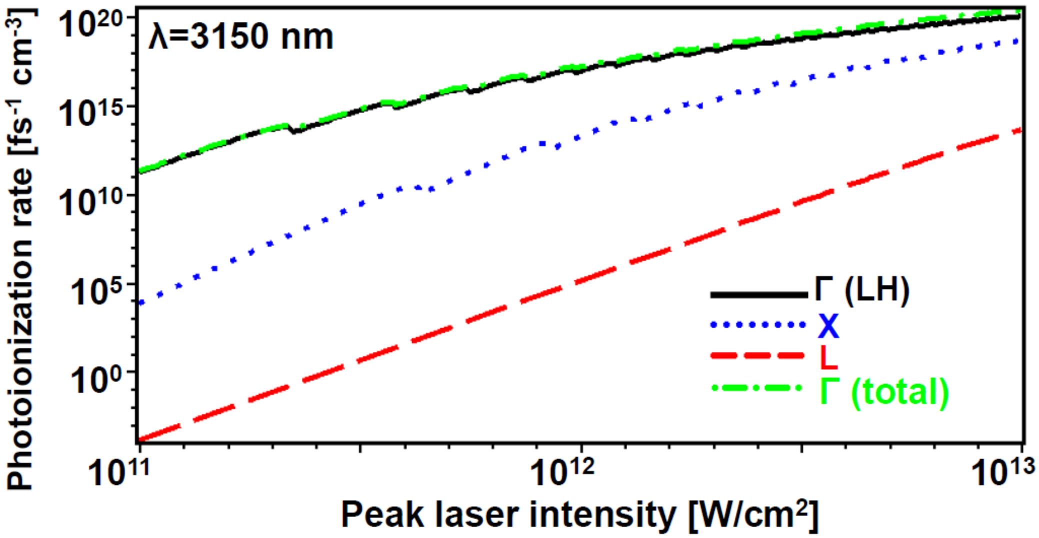

B

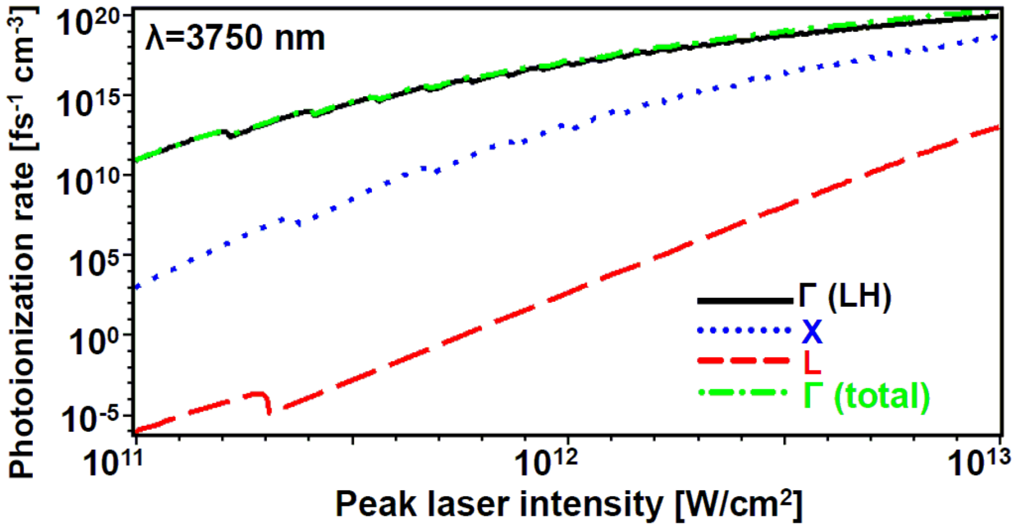

C

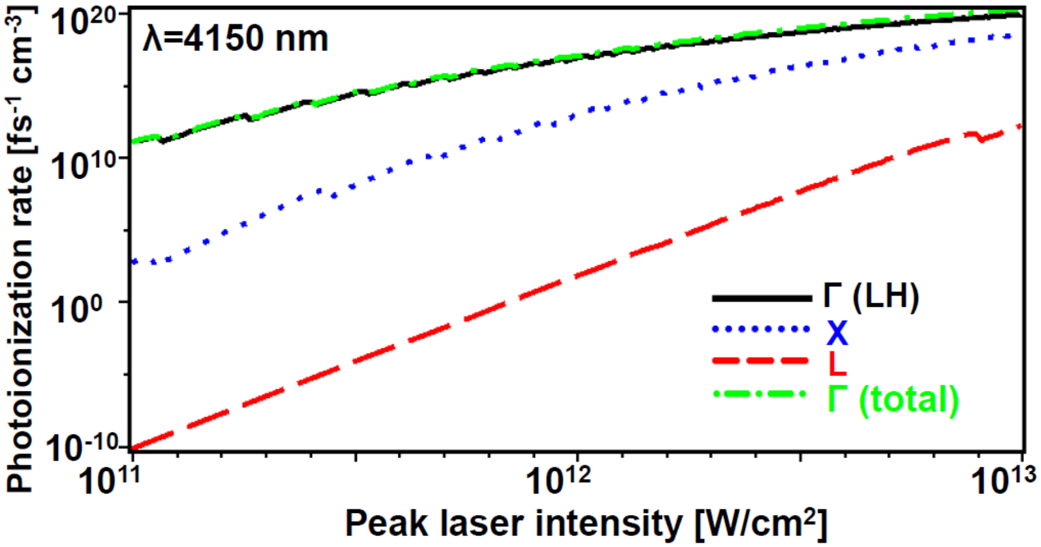

D

Figure S6: Intensity scaling of the photoionization rate for the direct inter-band transitions at the Γ (black and green), L (red dashed), and X (blue dotted) points of the Brillouin zone estimated at 2750 nm (A), 3150 nm (B), 3750 nm (C), and 4150 nm (D) wavelengths. For the Γ point, there are shown the contribution made by the light-hole valence band (black solid) and the total contribution by the three valence bands (green dash-dotted).

Intensity scaling of the transitions rates from the light-hole valence band to the Γ point of conduction band and to the virtual states V1 and V2 (at free-electron effective mass)

The evaluations of the electron transitions to the virtual states V1 and V2 (see Fig. 6A for the defect notation) are done for illustrative purposes. Since effective electron mass and energy-momentum relations associated with the virtual states are not known, the evaluations are done under some reasonable assumptions: parabolic energy-momentum relation and free-electron mass as the effective mass. Intensity scaling of the transition rates is depicted in Fig. S7. There are also checked two more options for the effective mass: a) the virtual-state effective masses are the same as the LH or HH effective mass (Fig. S8: A and B); and b) the virtual-state mases are the same as conduction-band effective masses of the X and L valleys (Fig. S8: C and D).

For the direct transitions at the Γ point of the Brillouin zone, there are evaluated the total contribution of all the three valence bands and the specific contribution of the LH valence band. In all considered cases, the rate of the transitions to the virtual states is at least 1-2 orders of magnitude larger than the rate of the direct inter-band transitions to the conduction band. That conclusion is employed for the estimations of Eq. (4) of the main text.

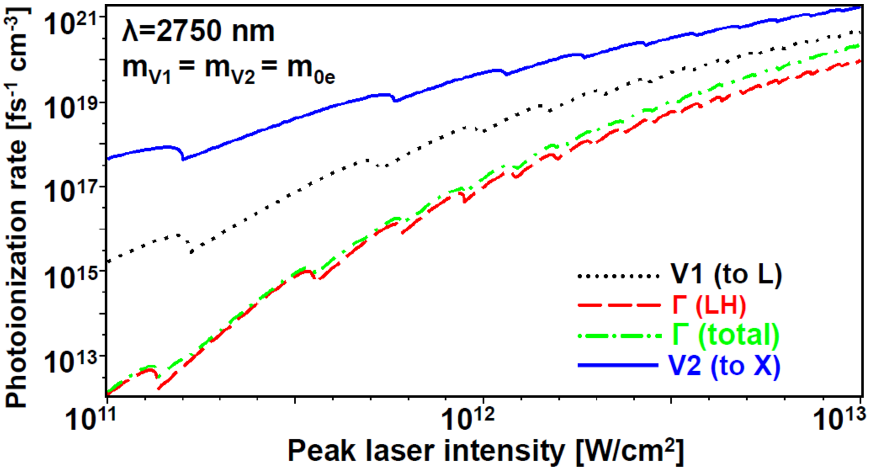

A

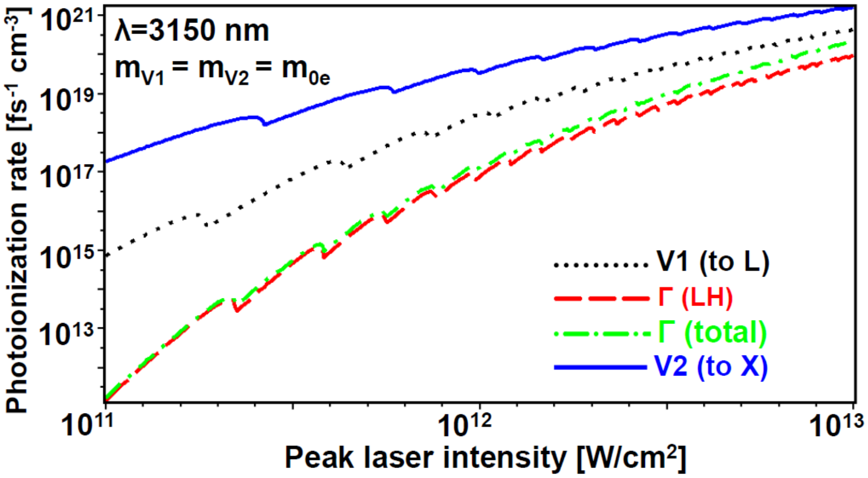

B

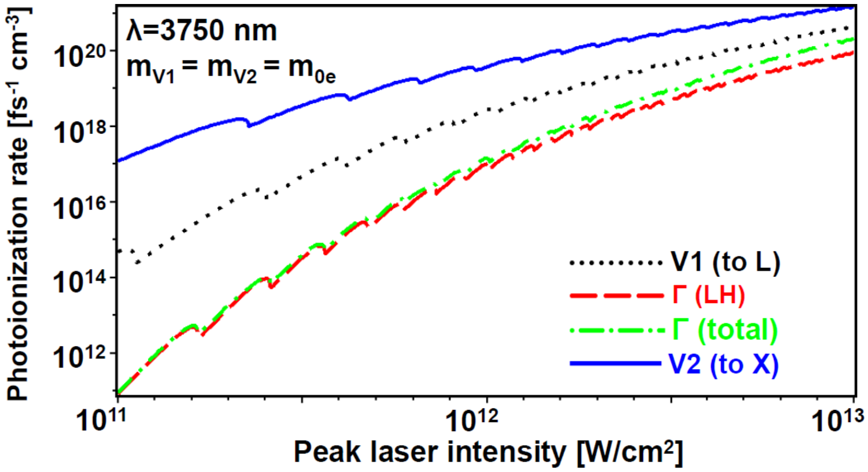

C

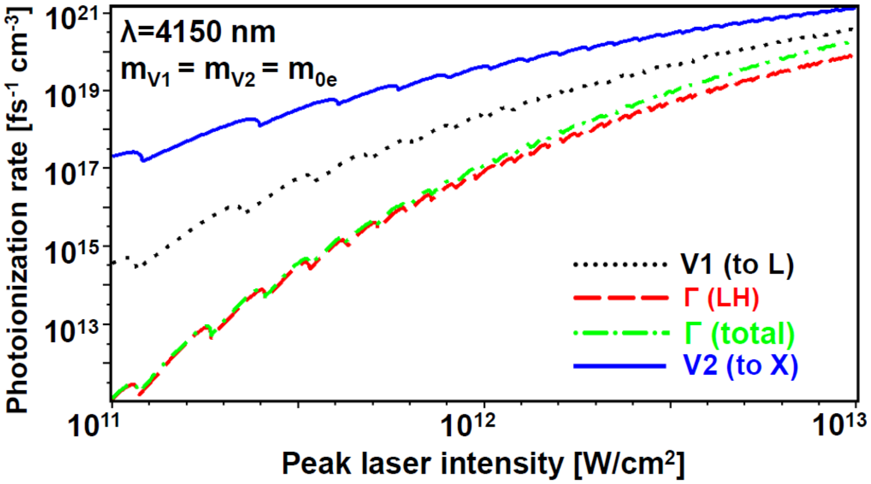

D

Figure S7: Intensity scaling of the transitions rates from the light-hole valence band to the virtual state V1 (black dotted), virtual state V2 (blue solid), and conduction band (red dashed) at the Γ point of the Brillouin zone for electric field directed parallel to the <111> crystal direction. The total rate of the transitions from three valence bands (green dash-dotted) is also shown. The rates are plotted for laser wavelengths 2750 nm (A); 3150 nm (B); 3750 nm (C); and 4150 nm (D). Effective electron mass of any of the virtual states is the free-electron mass.

Intensity scaling of the transitions rates from the light-hole valence band to the Γ point of conduction band and to the virtual states V1 and V2 (at LH-valence-band and conduction-band values of effective mass)

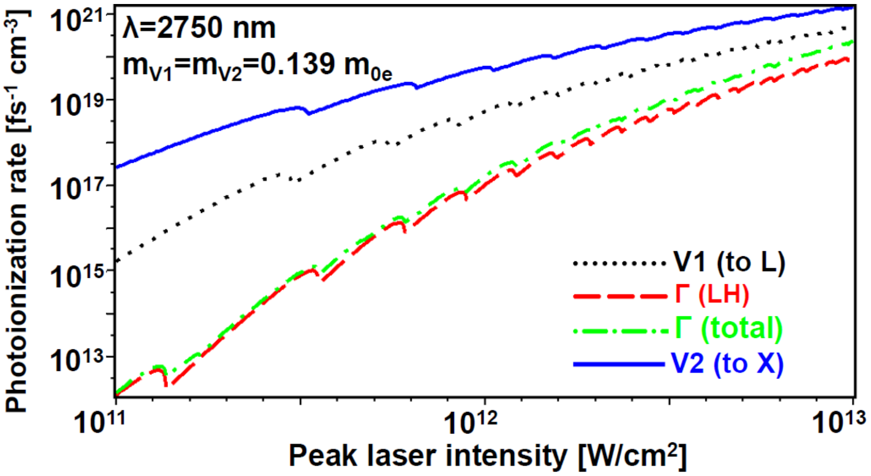

A

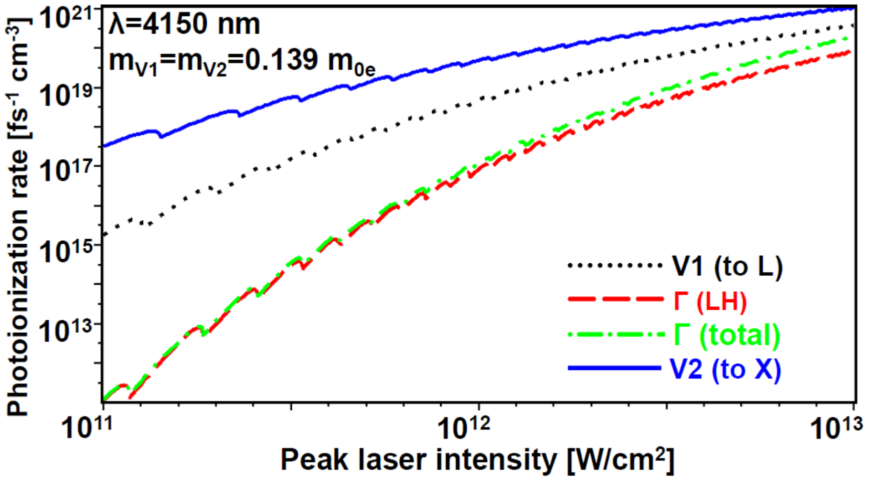

B

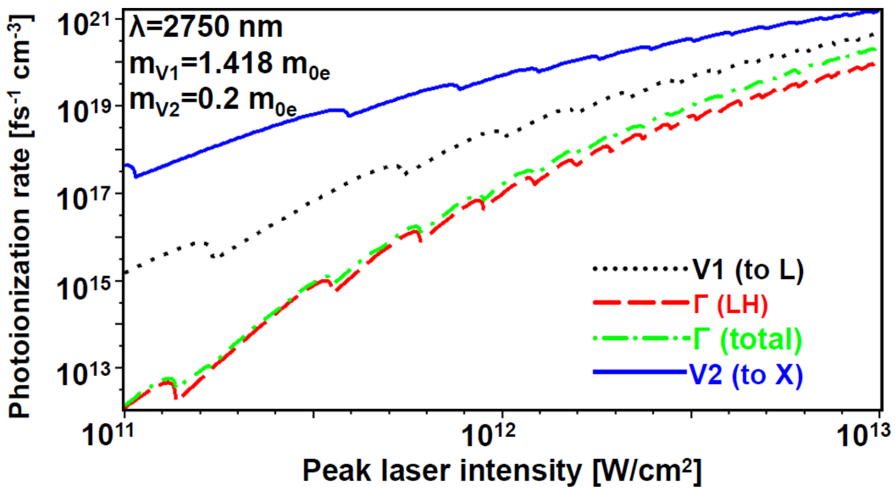

C

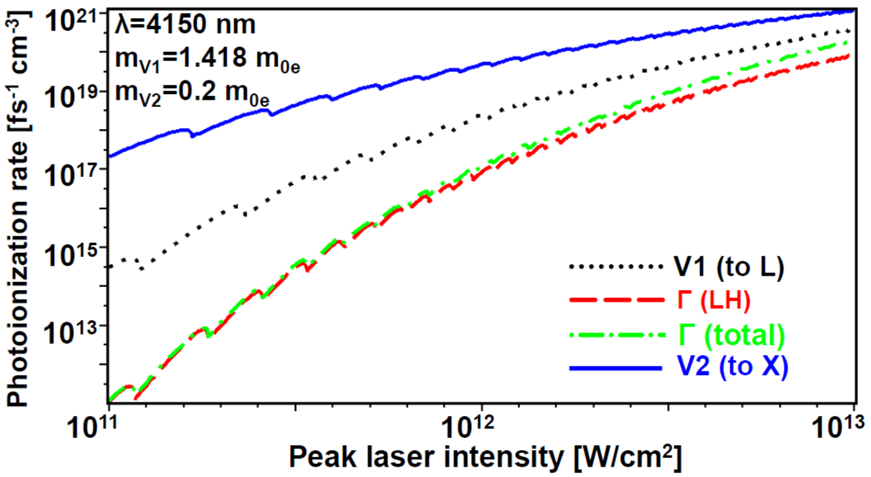

D

Figure S8: Intensity scaling of the transitions rates from the light-hole valence band to the virtual state V1 (black dotted), virtual state V2 (blue solid), and conduction band (red dashed) at the Γ point of the Brillouin zone for electric field directed parallel to the <111> crystal direction. The total rate of the transitions from three valence bands (green dash-dotted) is also shown. The rates are plotted for laser wavelengths 2750 nm (A, C) and 4150 nm (B, D). Effective electron masses of the virtual states are: I) the conduction-band effective mass (0.139 of the free-electron mass) for the both virtual states (A and B); and II) 1.418 of the free electron mass for V1, and 0.2 of the free electron mass for V2 (C and D correspondingly).

5. Time-domain simulations with the Keldysh formula

Additional implementation details of our simulation code is described in Appendix B of Ref. [8]. The simulation code is based on the Keldysh formula for the photoionization rate as described in section 4 above. The electric field envelope of the pulse, ionization rate, and carrier density are calculated within the material at the surface, for an array of input intensities and wavelengths for each time step. For each input intensity and wavelength, the total free-carrier density produced by a single pulse is compared against the free-carrier density for ultrafast melting defined as 10% of total valence-band population prior to laser action. Simulation parameters are as follows: wavelength step size is 20 nm, intensity step size is 10 GW/cm^2, and time step size is 8 fs with an 800 fs range. By utilizing the Keldysh formula above, we ignore all fast variations in the electric field. Note that for $\lambda = 2.5\mu m \ \frac{1}{\nu} \approx 8$ fs; we avoid sampling sub-cycle dynamics in our simulation to remain consistent with the Keldysh formula.

Representative results utilizing the Keldysh formula applied to one valence band (LH) are shown in Fig. S9. Fig. S9 demonstrates the steps involved in the simulation which models the temporal dynamics of the electric field in the medium (Fig. S9a.), then evaluates the Keldysh ionization rates from the field (Fig. S9b.), and finally evaluates the free-carrier density as a function of time (Fig. S9c.). For each input peak intensity, this process is repeated and a final free-carrier density produced by the entire pulse is recorded (Fig. S9d.). The peak intensity which results in the final free-carrier density matching the ultrafast melt condition discussed in the main text is considered as the LIDT intensity. Note that the photon-dominated energy deposition as described by the simulations based on the Keldysh formula completely fails, overshooting the experimental results by about one order of magnitude.

Figure S10 demonstrates changes to the simulation which led to a several times lower LIDT intensity (solid line in Fig. 5b of the main text), but still overshoot the experimental results and fail to capture the observed trend. The electric field is simulated as before (Fig. S10a.), but now we include all three valence bands (light hole, heavy hole, and split off) tracked separately as shown in Fig. S10 b-d. The population factor is multiplied by the rate to ensure that no valence band can individually exceed 100% depletion. Carrier recombination (~100 ps [9]), carrier-lattice thermalization (~260 fs [9]), and the optical Kerr effect ($n_2(\lambda=1.5\ \mu m) \approx 0.06 \frac{cm^2}{TW}$ [10]) are neglected here.

Results of time-domain simulation with the Keldysh formula

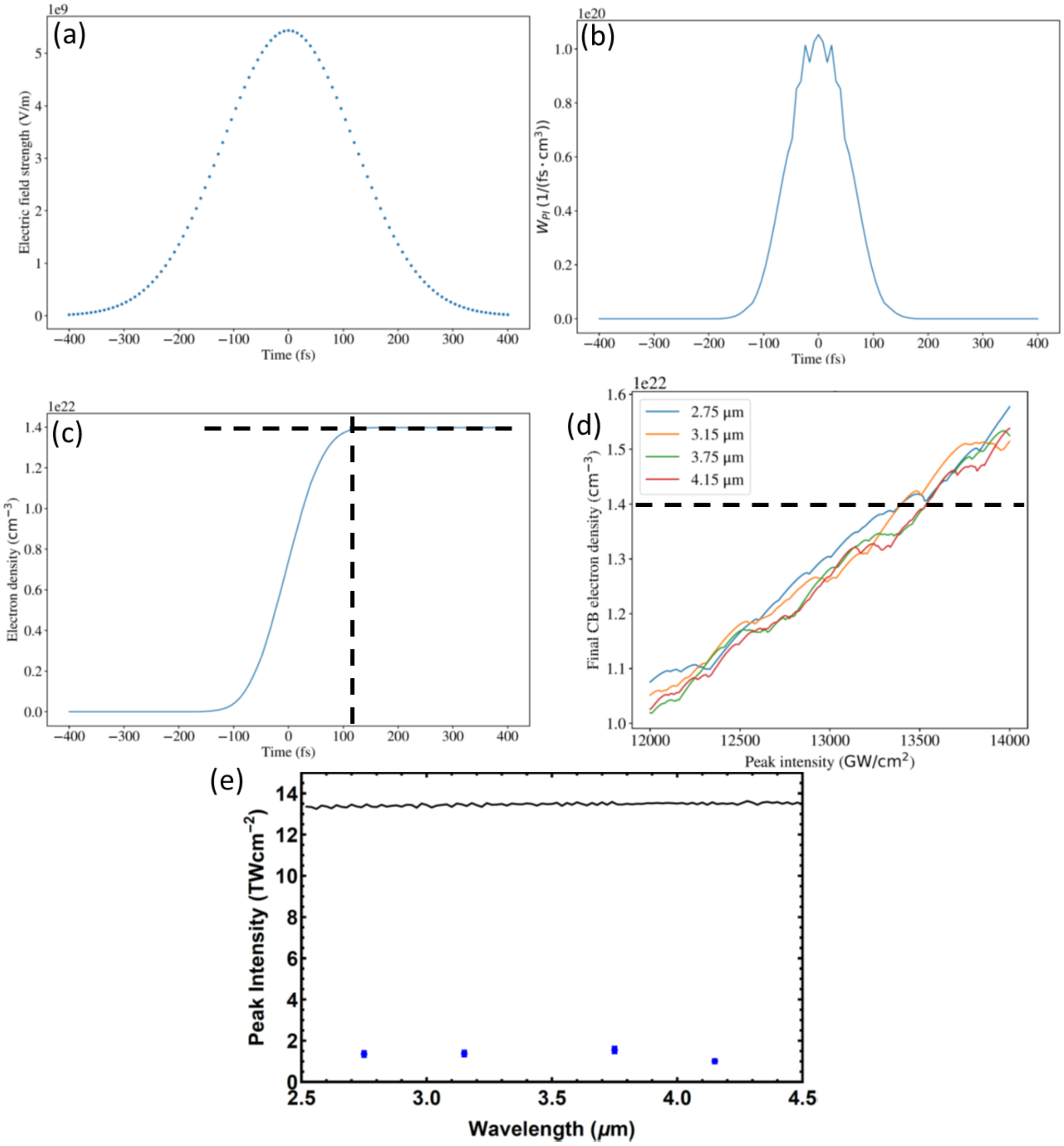

Figure S9: Results of time-domain single-valence-band simulation with the Keldysh formula. For (a)-(c) λ=3.15μm and the peak intensity I_0=13.5 TW/cm^2, equivalent to the simulated LIDT intensity. (a) Simulation input: electric field envelope inside the material vs time. (b) Photoionization rate dynamics (Keldysh equation numerical calculation). (c) Dynamics of the excited carrier density, which saturates to the LIDT threshold carrier density by a certain time in the simulation (denoted by dotted lines). (d) Final excited carrier density as a function of input peak intensity, for four distinct wavelengths denoted by the inset. The horizontal black dotted denotes the LIDT excited carrier density criterion. (e) LIDT intensity data is extracted from plots like (d) for many wavelengths. Points represent the experimental data; solids line – the simulation results with the one-valence-band Keldysh formula.

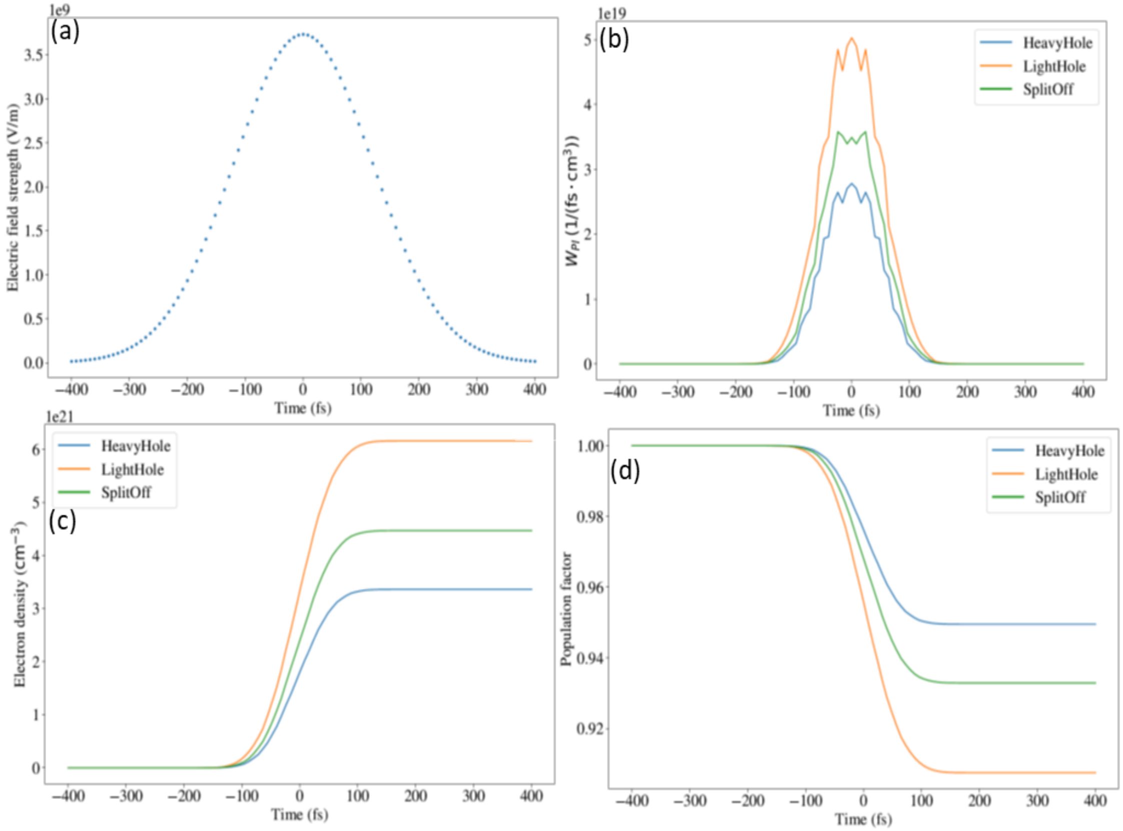

Figure S10: Keldysh photoionization simulation with multiple valence bands and valance band depletion. For (a)-(d) λ=3.15μm and the peak intensity I_0=9.74 TW/cm^2, equivalent to the simulated LIDT intensity. (a) Electric field amplitude inside the material. (b) Keldysh photoionization rate. The rate of each valence band is tracked separately (see notation in the inset). (c) Dynamics of free-carrier density; contribution from each valence band are depicted separately. The total contribution (sum from all bands) is used for evaluation of the LID threshold. (d) The time variations of the valence-band depletion factor shown for each valence band. It multiplies the corresponding terms of a rate equation and characterizes the laser-induced de-population of each valence band due to the inter-band transitions. For any individual valence band, 100% depletion cannot be achieved.

6. Simulations with the Gamaly model

The Gamaly model [8, 11] is a two-temperature model assuming the formation of a plasma early in the pulse. Energy absorbed by this plasma is calculated in a 1D approximation by assuming a high collision rate/ideal-gas dense plasma model: $\Gamma \approx \omega_p \gg \omega$ where Γ is the Drude collision rate [8], ω_p is the plasma frequency, and ω is the laser driving frequency. Conservation of energy is applied as follows: the mean thermal energy deposited into the material must be enough to (1) produce an electron-hole pair and (2) overcome the lattice binding energy. It is assumed that the laser-generated free electrons are emitted if the energy absorbed by the free carriers exceeds the sum of band gap and electron affinity. A thin surface layer of ions can then be removed (ablation) due to generation of localized positive charge via the electron emission. Since the final surface temperature linearly scales with the incident laser pulse intensity [11], a fluence threshold for ablation can be estimated [8, 11] as follows:

$$\Phi = \frac{3}{4}(\Delta + E_{\mathrm{b}})\frac{l_{\mathrm{s}} N_{\mathrm{CB}}}{A} \qquad (S13)$$

where N_{CB} is the conduction band electron density, Δ is the bandgap, E_b is the binding energy for atoms/ions of a crystal lattice, l_s=c/(ωIm(n)), c is the speed of light, n is the refractive index, and ω is the laser frequency, and the absorption coefficient at normal incidence [11]:

$$A_{s/p} = 1 - R \approx \left|\frac{\pm\cos\theta \mp \sqrt{\mathrm{n}^2 - \sin^2\theta}}{\cos\theta + \sqrt{n^2 - \sin^2\theta}}\right|^2 \qquad (S14)$$

where θ is the angle of incidence. Next, we note that S13 can be rewritten:

$$\Phi = \frac{3}{4}(\Delta + E_{\mathrm{b}} + \chi_{\mathrm{e}})\frac{l_{\mathrm{s}} N_{\mathrm{CB}}}{A} \qquad (S15)$$

where χ_e is the electron affinity, the energy required to promote an electron from the lowest conduction-band bottom to the vacuum states. This energy cost correction (Equation S15) to the original Gamaly equation (Equation S13) is physically realizable for the above mentioned damage mechanism, where charge separation is necessary [8].

The Gamaly model assumption that N_{CB} is equal to the atomic number density, is not a reasonable assumption based on Keldysh photoionization results [8]. Instead, we make the more reasonable choice for N_{CB}: to be 10% [8] of the valence band electron density, the threshold criterion for ultrafast melting [12].

The Gamaly model calculation of the main text used the following parameters: 200 fs FWHM pulse duration, 31 degrees angle of incidence, p-polarization, 2-4.5 um central wavelength with 100 steps. Both direct and indirect bandgaps were assumed for comparison. The full complex index of refraction is used to calculate the absorbance.

7. Microscopic criteria of damage/ablation threshold in semiconductors

Several microscopic criteria of ultrafast-ablation and ultrafast LID threshold are available in publications [11-16]. Here, we summarize some quantitative characteristics and basic formulae of the major LID/ablation criteria. Relevance of each particular criterion to our mid-IR ultra-fast laser experiments is discussed in the main text.

1) Critical absorbed energy (CAE) criterion considers deposition of certain minimum amount of absorbed laser-pulse energy per each laser-generated free electron within some laser-solid interaction volume V_{in} to produce ions and initiate departure of the ions from an ablated surface [11]. The original version of this criterion (see Eq. (24) of Ref. [11]) estimates the CAE via a sum of band gap Δ (the minimum energy required to produce a free electron in the conduction band) and binding energy E_b required for an ion to depart from its position at the surface:

$$E_A = (\Delta + E_{\mathrm{b}})N_{\mathrm{CB}}V_{in} \qquad (S16)$$

However, this balance of the absorbed energy misses the amount of electron's energy required to remove the electron from the surface for production of the ions. This process can be considered as electron emission from the surface. Correspondingly, with the accuracy of the original model of Ref. [11], the minimum electron-escape energy is estimated as electron affinity χ_e in dielectrics or semiconductors. The modified CAE criterion then reads as follows:

$$E_A = (\Delta + E_{\mathrm{b}} + \chi_e)N_{\mathrm{CB}}V_{in} \qquad (S17)$$

The sum of the free-carrier energy $E_b + \chi_e$ is from 5 to 7 eV for a majority of typical non-metal crystals [2]. Therefore, the CAE criterion considers excitation of the laser-generated free carriers to rather high average energy of a few eV.

2) Plasma-frequency critical electron-hole plasma density (PF CED) is one the most frequently utilized criterion of LID or ablation threshold [15]. It estimates the minimum free-carrier density by equating laser frequency ω and critical plasma frequency ω_{ep} based on the Drude model of free electrons in metals [2]. According to that model, the condition $\omega_{ep} = \omega$ corresponds to a strong reduction of transparency of the free-carrier plasma [2] that prevents propagation of laser radiation through the sub-surface layer where the critical-density plasma is generated. The PF CED criterion frequently considers the free electrons [15] at the very bottom of the conduction band with effective electron mass m_{CB} and reads as follows [2, 15]:

$$N_P = \frac{\varepsilon_0 m_{CB}\omega^2}{e^2} \qquad (S18)$$

where e is electron charge, and ε_0 is electric constant. However, if contribution of the laser-generated free holes with effective mass m_{VB} cannot be neglected (that is the case for typical semiconductors), the effective electron mass of Eq. (18) must be replaced with electron-hole reduced effective mass m_{eff}. For direct-gap crystals with an isotropic conduction-band valley at the center of the first Brillouin zone, the reduced effective mass is calculated as follows:

$$\frac{1}{m_{eff}} = \frac{1}{m_{CB}} + \frac{1}{m_{VB}} \qquad (S19)$$

In case of crystals with conduction-band valleys located out of the Brillouin-zone center, the reduced electron-hole mass m_{eff} of Eq. (S19) is to be replaced with optical effective mass of the free carriers evaluated via their longitudinal and transverse effective masses in the conduction-band valleys (see, for example, evaluation of the optical effective mass for silicon in Ref. [13]).

This PF CED criterion does not include any requirements for average free-carrier energy, but the usual approximations utilized for evaluation of the critical electron density with Eq. (S18), e. g., the approximation of constant effective mass, assume the free electrons populate the bottom part of a conduction-band valley and are not substantially promoted to higher conduction-band states. Also, the PF CED criterion is essentially based on the assumption that the optical response is produced by a quasi-equilibrium free-carrier plasma, which particles have experienced enough collisions to arrive at a quasi-equilibrium state of the entire free-carrier ensemble.

3) Plasma-resonance critical electron-hole plasma density (PR CED) criterion considers the free-carrier density that reduces the real part of transient optical response to almost zero [13, 14]. It is also referred to as *epsilon-near-zero (ENZ)* criterion [14]). Evaluation of the PR CED substantially depends on a specific model of permittivity. Following the commonly accepted approximations [13, 14], we assume that the real part of the permittivity includes reduction of polarization response from valence electrons due to inter-band electron transitions and negative response of the laser-generated free carriers:

$$\varepsilon_{Re} = \varepsilon_g(\omega) - [\varepsilon_g(\omega) - 1]\frac{N_e}{N_0} - \frac{N_e}{N_P}\frac{\omega^2\tau_D^2}{1+\omega^2\tau_D^2} \qquad (S20)$$

where N_0 is total valence-band electron density, N_e is the time-dependent density of laser-generated conduction-band electrons, ε_g is the real part of permittivity at laser wavelength prior to laser action, τ_D is the Drude-model collision time [2, 13], and N_P is given by Eq. (S18). Then, the PR CED criterion reads as follows:

$$N_{PR} = \frac{N_P\varepsilon_g(\omega)}{[\varepsilon_g(\omega) - 1]\frac{N_P}{N_0} + \frac{\omega^2\tau_D^2}{1+\omega^2\tau_D^2}} \qquad (S21)$$

The frequently cited criteria of plasma-resonance density and plasma resonance [13, 14]:

$$N_{PR} = \varepsilon_g(\omega)N_P; \;\; \omega_p = \sqrt{\varepsilon_g(\omega)}\omega \qquad (S22)$$

where time-dependent plasma frequency reads as follows [13]:

$$\omega_P^2 = \frac{N_e e^2}{\varepsilon_0 m_{eff}} \qquad (S23)$$

are derived from Eq. (S21) by assuming low free-carrier density and low collision rate:

$$N_P \ll N_0; \;\; \omega\,\tau_D \gg 1 \qquad (S24)$$

Eq. (S22) suggests that NPR is of the same order of magnitude as NP of Eq. (S18) in dielectrics with characteristic values of permittivity varying from 2 to 4. In typical semiconductors, the real part of permittivity is of the order of 10 [13], and the PR CED of Eq. (S22) is an order of magnitude larger than the plasma density of Eq. (S18). It is remarkable that the epsilon-near-zero condition attributed to this PR CED increases reflectivity R to almost 1 if imaginary part of the permittivity ε_{Im} is small compared to 1 at the plasma density given by Eq. (21):

$$R = 1 - 2\sqrt{2\varepsilon_{Im}} \qquad (S25)$$

Similar to PF CED, the PR CED criterion does not involve requirements on average free-carrier energy and is frequently attributed to the approximations characteristic of low-energy electrons at the very bottom part of a conduction-band valley. Also, the PR CED criterion assumes that the optical response is produced by a quasi-equilibrium free-carrier plasma, for which particles have

experienced multiple collisions to arrive at a quasi-equilibrium state.

4) Epsilon-near-one (ENO) criterion for free-carrier density is sometimes referred as another criterion for evaluation of ablation threshold [14]. It is derived by equating real part of permittivity to 1.0 and reads as follows for the cited above model of permittivity:

$$N_{PR} = \frac{N_P\left[\varepsilon_g(\omega) - 1\right]}{\left[\varepsilon_g(\omega) - 1\right]\frac{N_P}{N_0} + \frac{\omega^2\tau_D^2}{1+\omega^2\tau_D^2}} \qquad (S26)$$

Under the conditions of Eq. (S24), it reduces to the usual form utilized, e. .g., in Ref. [14]:

$$N_{PR} = \left[\varepsilon_g(\omega) - 1\right]N_P \qquad (S27)$$

Similar to the criteria PF CED and PR CED, this criterion does not require high energy of the conduction-band electrons and assumes quasi-equilibrium optical response of the free-carrier plasma. Also, the epsilon-near-one condition reduces reflectivity of the crystal to almost zero if imaginary part of the permittivity ε_{Im} is small compared to 1 at the plasma density of Eq. (26):

$$R = 0 + \frac{\varepsilon_{Im}}{16} \qquad (S28)$$

5) Crystal-lattice instability critical electron-hole density (LI CED) considers softening of phonons I. e., laser-induced modification of phonon frequency) as a signature of a process of developing crystal-lattice instability induced by increase of conduction-band population [13, 16]. There is no analytical relation for this criterion, but ab-initio simulations and theoretical estimations [13, 16] suggest that phonon softening takes place if approximately 5 to 10% of valence-band electrons are promoted to the conduction band by laser radiation. In case of silicon, this criterion corresponds to generation of approximately 10^{21} – 10^{22} free carriers per cubic cm. Those simulations and estimations assume low energy of the conduction-band electrons [16].

6) Plasma-instability critical free-carrier plasma density (PI CED) criterion was originally proposed to explain laser-induced ultrafast annealing of semiconductors [13]. Experimental measurements of laser-produced free carriers in silicon [13] suggest that the PI CED is several times higher than the LI CED and exceeds the 10% figure of merit of the LI CED criterion. Correspondingly, the free-carrier density of this criterion exceeds 10^{22} 1/cm^3. In contrary to LI CED, this criterion may involve intra-band excitation of the conduction-band electrons to the energies a few eV above the conduction-band bottom. However, this point has received no substantial study so far, and the role of average free-electron energy is not clear. Also, the density estimated from reflectivity measurements of Ref. [13] is affected by assumptions about electron parameters, e. g., Drude-model collision time and effective electron mass. In particular, those parameters were assumed to be energy independent in Ref. [13].

Supplementary references:

[1] Keldysh, L.V. Ionization in Field of a Strong Electromagnetic Wave. *Soviet Physics Jetp-Ussr* **20**, 1307-& (1965).

[2] Ashcroft, N. W., Mermin, D. N. in *Solid state physics* (Holt, Rinehart and Winston, New York, 1976).

[3] Cohen, M. L., Bergstresser T. K. Band Structure and Pseudopotential Form Factors for Fourteen Semiconductors of the Diamond and Zinc-blende Structures, *Phys. Rev.* **141** (2), 789-796 (1966).

[4] Chelikowsky, J. R., Cohen, M. L. Nonlocal pseudopotential calculations for the electronic structure of eleven diamond and zinc-blende semiconductors. *Phys. Rev. B* **14** (2), 556-582 (1976).

[5] Gruzdev, V. Fundamental mechanisms of laser damage of dielectric crystals by ultrashort pulse: ionization dynamics for the Keldysh model. *Optical Engineering* **53**, 122515 (2014).

[6] Gruzdev, V. E., Komolov, V. L., Przhibel'skii, S. G. Ionization of nanoparticles by supershort moderate-intensity laser pulses. *Journal of Opt. Technol.* **81**(5), 256-261 (2014).

[7] Shcheblanov, N. S. Numerical study of femtosecond laser interactions with dielectric materials: application to the definition of damage threshold of optical components (Ph.D. Thesis, 2013); http://www.theses.fr/2013STET4029.

[8] Austin, D. R. Semiconductor surface modification using mid-infrared, femtosecond laser pulses. (The Ohio State University, 2017).

[9] Sabbah, A. & Riffe, D. Femtosecond pump-probe reflectivity study of silicon carrier dynamics. *Physical Review B* **66**, 165217 (2002).

[10] Tsang, H. *et al*. Optical dispersion, two-photon absorption and self-phase modulation in silicon waveguides at 1.5 mu m wavelength. *Appl. Phys. Lett.* **80**, 416-418 (2002).

[11]. Gamaly, E., Rode, A., Luther-Davies, B. & Tikhonchuk, V. Ablation of solids by femtosecond lasers: Ablation mechanism and ablation thresholds for metals and dielectrics. *Phys Plasmas* **9,** 949-957 (2002).

[12]. Austin, D. R., Kafka, K. R. P., Lai, Y. H., Wang, Z., Blaga, C. I. & Chowdhury, E. A. Femtosecond laser damage of germanium from near- to mid-infrared wavelengths. *Opt. Lett.* **43,** 3702-3705 (2018).

[13] Sokolowski-Tinten, K. & von der Linde, D. Generation of dense electron-hole plasmas in silicon. *Physical Review B* **61**, 2643-2650 (2000).

[14] Gamaly, E. G., Rode, A. V., Ultrafast re-structuring of the electronic landscape of transparent dielectrics: new material states (Die-Met). *Appl. Phys. A* **124**, 278 (2018).

[15] Balling, P. & Schou, J. Femtosecond-laser ablation dynamics of dielectrics: basics and applications for thin films. *Rep. Prog. Phys.* **76**, 036502 (2013).

[16] Stampfli, P. & Bennemann, K. Dynamic Theory of the Laser-Induced Lattice Instability of Silicon. *Physical Review B* **46**, 10686-10692 (1992).
